# Association Between Magnitude of Differential Blood Pressure Reduction and Secondary Stroke Prevention: A Meta-analysis and Meta-Regression

**DOI:** 10.1001/jamaneurol.2023.0218

**Published:** 2023-03-20

**Authors:** Chia-Yu Hsu, Jeffrey L. Saver, Bruce Ovbiagele, Yi-Ling Wu, Chun-Yu Cheng, Meng Lee

**Affiliations:** 1 Department of Neurology, Chang Gung University College of Medicine, Chang Gung Memorial Hospital Chiayi Branch, Puzi, Taiwan; 2 UCLA Stroke Center, Department of Neurology, University of California, Los Angeles, Los Angeles; 3 Department of Neurology, University of California, San Francisco, Los Angeles; 4 Institute of Population Health Sciences, National Health Research Institutes, Miaoli County, Taiwan; 5 Department of Neurosurgery, Chang Gung University College of Medicine, Chang Gung Memorial Hospital Chiayi Branch, Puzi, Taiwan

## Abstract

**Importance:**

The degree to which more intensive blood pressure reduction is better than less intensive for secondary stroke prevention has not been delineated.

**Objective:**

To perform a standard meta-analysis and a meta-regression of randomized clinical trials to evaluate the association of magnitude of differential blood pressure reduction and recurrent stroke in patients with stroke or transient ischemic attack (TIA).

**Data Sources:**

PubMed, Embase, the Cochrane Central Register of Controlled Trials, and ClinicalTrials.gov were searched from January 1, 1980, to June 30, 2022.

**Study Selection:**

Randomized clinical trials that compared more intensive vs less intensive blood pressure lowering and recorded the outcome of recurrent stroke in patients with stroke or TIA.

**Data Extraction and Synthesis:**

The Preferred Reporting Items for Systematic Reviews and Meta-analyses (PRISMA) reporting guideline was used for abstracting data and assessing data quality and validity. Risk ratio (RR) with 95% CI was used as a measure of the association of more intensive vs less intensive blood pressure lowering with primary and secondary outcomes. The univariate meta-regression analyses were conducted to evaluate a possible moderating effect of magnitude of differential systolic blood pressure (SBP) and diastolic blood pressure (DBP) reduction on the recurrent stroke and major cardiovascular events.

**Main Outcomes and Measures:**

The primary outcome was recurrent stroke and the lead secondary outcome was major cardiovascular events.

**Results:**

Ten randomized clinical trials comprising 40 710 patients (13 752 women [34%]; mean age, 65 years) with stroke or TIA were included for analysis. The mean duration of follow-up was 2.8 years (range, 1-4 years). Pooled results showed that more intensive treatment compared with less intensive was associated with a reduced risk of recurrent stroke in patients with stroke or TIA (absolute risk, 8.4% vs 10.1%; RR, 0.83; 95% CI, 0.78-0.88). Meta-regression showed that the magnitude of differential SBP and DBP reduction was associated with a lower risk of recurrent stroke in patients with stroke or TIA in a log-linear fashion (SBP: regression slope, −0.06; 95% CI, −0.08 to −0.03; DBP: regression slope, −0.17; 95% CI, −0.26 to −0.08). Similar results were found in the association between differential blood pressure lowering and major cardiovascular events.

**Conclusions and Relevance:**

More intensive blood pressure–lowering therapy might be associated with a reduced risk of recurrent stroke and major cardiovascular events. These results might support the use of more intensive blood pressure reduction for secondary prevention in patients with stroke or TIA.

## Key Points

**Question:**

How much better is more blood pressure reduction vs less blood pressure reduction for secondary stroke prevention?

**Findings:**

In this meta-analysis and meta-regression that included 10 randomized clinical trials comprising 40 710 patients with stroke or transient ischemic attack, the risk of recurrent stroke was 8.4% with more intensive blood pressure lowering vs 10.1% with less intensive or no blood pressure lowering, a statistically significant difference. The greater the amount of differential blood pressure reduction, the greater the amount of reduction risk of recurrent stroke.

**Meaning:**

This study suggests that more intensive differential blood pressure–lowering therapy may be beneficial for secondary stroke prevention.

## Teaser

This meta-analysis and meta-regression of randomized clinical trials evaluates the association of magnitude of differential blood pressure reduction and recurrent stroke in patients with stroke or transient ischemic attack.

## Introduction

Hypertension is a major risk factor for recurrent stroke in patients with ischemic and hemorrhagic stroke or transient ischemic attack (TIA), and blood pressure lowering is therefore a guideline-recommended strategy to prevent recurrent stroke. Indeed, recurrent stroke has declined substantially over decades, with improved blood pressure control as a leading cause.^1^

However, most individual randomized clinical trials have not significantly showed that blood pressure–lowering therapy reduces recurrent stroke in patients with stroke or TIA. Relatively small magnitude of blood pressure reduction and relatively few participants and inadequate statistical power in single randomized clinical trials may have attenuated signals of efficacy.^2,3^ Furthermore, some trials^2,4^ compared antihypertensive therapy with placebo, while other trials^3,5^ compared a lower systolic blood pressure (SBP) target with a higher SBP-lowering target. Pooling of data from these 2 types of trials is needed to clarify the association between degree of increased blood pressure lowering attained with therapy and secondary stroke prevention.

Therefore, we performed a systematic review and meta-analysis of all relevant randomized clinical trials to evaluate the association between magnitude of differential blood pressure reduction and risk of recurrent stroke and performed a meta-regression to clarify whether larger differential blood pressure reduction magnitude was monotonically associated with a lower recurrent stroke risk in patients with stroke or TIA.

## Methods

The Preferred Reporting Items for Systematic Reviews and Meta-analyses (PRISMA) reporting guideline was used for abstracting data and validity of this meta-analysis.^6^ The protocol was registered with PROSPERO (CRD42022309056).

### Search Methods and Resources

We searched PubMed, Embase, the Cochrane Central Register of Controlled Trials, and the clinical trial registry maintained at ClinicalTrials.gov from January 1, 1980, to June 30, 2022, using the following terms: stroke or cerebrovascular accident or brain vascular accident or cerebral infarct or cerebrovascular disorder or intracranial vascular disease or cerebrovascular disease or cerebrovascular occlusion or transient ischemic attack AND antihypertensive or blood pressure lowering or blood pressure reduction AND recurrent or secondary prevention or previous or prior or history. We restricted the search to studies in humans and randomized clinical trials and did not apply language restrictions. We also reviewed prior meta-analyses^7,8,9^ to identify additional trials. Two investigators (C.-Y.H. and Y.-L.W.) independently screened and identified potential trials, and discrepancies were resolved by discussion with a third investigator (M.L.).

### Study Selection and Data Extraction

Criteria for inclusion of a study were as follows: (1) the study design was a randomized clinical trial; (2) all or an identifiable subset of participants had a history of stroke or TIA; (3) the study evaluated more intensive vs less intensive blood pressure–lowering therapy, including the following possible comparisons: antihypertensive drug(s) vs placebo and a lower blood pressure target vs a higher blood pressure target; (4) recurrent stroke was reported as an end point; (5) the magnitude of SBP reduction between more intensive and less intensive blood pressure lowering was reported; and (6) treatment duration was of at least 1 year.

Criteria for exclusion of a study were as follows: (1) the study was published before 1980 because strategies for secondary stroke prevention, including antihypertensive drug use, were substantially different then^1^; (2) the study adopted only nonpharmaceutical approach, such as salt restriction or exercise, in the active treatment arm; (3) the study compared one antihypertensive drug vs another antihypertensive drug; (4) achieved SBP was higher in more intensive blood pressure–lowering group than less intensive blood pressure–lowering group after treatment; (5) more than 10% of patients were enrolled within 3 days after stroke; or (6) more than 10% of patients had end-stage kidney disease because of differential hemodynamic vulnerability of this disease. We extracted characteristics of each trial, which included patient age, sex, baseline blood pressure, number of patients in more intensive and less intensive blood pressure–lowering groups, duration of follow-up, magnitude of differential blood pressure reduction between more intensive and less intensive blood pressure–lowering groups, and number of recurrent stroke events and other outcomes in more intensive and less intensive blood pressure–lowering groups. Two investigators (C.-Y.H. and M.L.) independently abstracted the data and any discrepant judgments were resolved by joint discussion and by referencing the original report.

### Study Quality Assessment

Because all of the included studies were randomized clinical trials, the risk of bias (eg, selection bias, performance bias, detection bias, attrition bias, reporting bias, and other issues) of the included trials was assessed by the Cochrane risk-of-bias algorithm.^10,11^

### Outcomes

The primary outcome was recurrent stroke. The lead secondary outcome was major cardiovascular events. Major cardiovascular events were defined as the composition of nonfatal stroke, nonfatal myocardial infarction, and death from cardiovascular causes. Additional secondary outcomes were recurrent ischemic stroke, hemorrhagic stroke, fatal or disabling stroke, myocardial infarction, death from cardiovascular causes, death from any cause, and heart failure.

### Statistical Analysis

The analysis plan was performed on an intention-to-treat basis. We computed the fixed-effects estimate based on the Mantel-Haenszel method. Risk ratio (RR) with 95% CI was used as a measure of the association of more intensive vs less intensive blood pressure lowering with the primary and secondary outcomes. To explore the association between magnitude of differential blood pressure reduction and risk of recurrent stroke, analyses were performed for thresholds of SBP reduction of 4 mm Hg or lower, 5 mm Hg or lower, more than 5 mm Hg, more than 7 mm Hg, and more than 11 mm Hg, as well as magnitude of differential diastolic blood pressure (DBP) reduction of 2 mm Hg or lower, 3 mm Hg or lower, more than 3 mm Hg, and more than 4 mm Hg between more intensive vs less intensive blood pressure–lowering groups. The univariate meta-regression analyses were conducted with the fixed-effects model to evaluate a possible moderating effect of magnitude of differential SBP and DBP reduction on the recurrent stroke and major cardiovascular events. All *P* values were from 2-sided tests, and results were deemed statistically significant at *P* < .05. Heterogeneity was assessed by a *P* value determined by the use of χ^2^ statistics and *I*^2^ statistics, and *I*^2^ values of 0% to 29%, 30% to 49%, 50% to 74%, and 75% to 100% represent not important, moderate, substantial, and considerable inconsistency, respectively.^12^

The trim-and-fill method to identify and correct for funnel plot asymmetry arising from publication bias was used.^13^ A sensitivity test was conducted to identify any trial that might have exerted a disproportionate influence on the summary treatment effect on the primary outcome by removing each individual trial from the meta-analysis one at a time. Another sensitivity test was conducted by restricting analysis within trials with recurrent stroke being the primary outcome in the original trial design. Grading of Recommendations, Assessment, Development and Evaluations was used to evaluate summaries of evidence for the primary and secondary outcomes.^14,15^

Subgroup analyses of included trials were conducted according to different study characteristics: mean baseline SBP levels (≥150 mm Hg vs 140-149 mm Hg), mean achieved SBP levels in the more intensive and less intensive blood pressure–lowering groups (≥140 mm Hg vs 130 to <140 mm Hg vs <130 mm Hg), study duration (<3 years vs ≥3 years), sample size (<3000 vs ≥3000 patients), time interval from index stroke to randomization (within 6 months from stroke vs within 3-5 years from stroke), entry event (ischemic stroke vs hemorrhagic stroke), study design (antihypertensive drugs vs placebo and a lower blood pressure target vs a higher blood pressure target), definition of differential blood pressure reduction (mean difference throughout the studies vs other definitions), and antihypertensive drugs used in the more intensive treated arm (angiotensin-converting enzyme [ACE] inhibitors vs angiotensin receptor blockers vs β-blockers vs diuretics vs ACE inhibitors plus diuretics). Since the risk of recurrent ischemic and hemorrhagic stroke is greater in Asian populations with high blood pressure compared with several other groups around the world,^16^ we also conducted a subgroup analysis for Asians vs non-Asians. The Cochrane Collaboration’s Review Manager Software Package version 5.4 (RevMan) and Stata/SE version 15.1 (StataCorp LLC) were used for this meta-analysis and mete-regression.

## Results

We identified 21 full articles for detailed assessment, of which 11 did not meet the inclusion criteria; therefore, the final analysis included 10 randomized clinical trials (eFigure 1 in Supplement 1).^2,3,4,5,17,18,19,20,21,22^ The characteristics of the included trials are shown in Table 1.^2,3,4,5,17,18,19,20,21,22,23^ Overall, 40 710 patients (13 752 women [34%]; mean age, 65 years) with stroke or TIA were enrolled. The mean duration of follow-up was 2.8 years (range, 1-4 years). Among the 10 included trials, 6 compared antihypertensive drug(s) vs placebo or no antihypertensive therapy,^2,4,17,18,19,20^ and 4 compared a lower blood pressure target vs a higher blood pressure target.^3,5,21,22^ In the Perindopril Protection Against Recurrent Stroke Study (PROGRESS), 2 different active groups (perindopril alone and a combination of peridopril plus indapamide) were compared with placebo, and we analyzed separately perindopril alone vs placebo and perindopril plus indapamide vs placebo.^4^ Across all trials, the mean baseline SBP was 146 mm Hg and DBP was 85 mm Hg.

**Table 1.** Characteristics of Included Trials

Source	Population	Time interval from stroke to randomization	Intensity of BP lowering		Sample size, No.	Women, %	Mean, y		Primary outcome of original trial	BP at baseline, mm Hg	Definition of differential BP reduction	Magnitude of differential BP reduction, mm Hg
			More	Less			Age	Follow-up duration				
PAST-BP,^21^ 2016, UK	Stroke or TIA	NA	Lowering SBP to <130 mm Hg	Lowering SBP to <140 mm Hg	529	41	72	1	Change in SBP	143/80	Mean difference between the 2 groups throughout the study	2.9/1.6
PRoFESS,^2^ 2008, 35 countries	Ischemic stroke	Within 90 d	Telmisartan, 80 mg, daily	Placebo	20 332	36	66	2.5	Recurrent stroke	144/84	Mean difference between the 2 groups throughout the study	3.8/2
TEST,^18^ 1995, Sweden	Stroke or TIA	Within 3 wk	Atenolol, 50 mg, daily	Placebo	720	40	70	2.6	Death from any cause, nonfatal MI, and nonfatal stroke	161/89	Difference between the 2 groups after 1 mo of treatment	4/3
PROGRESS (single),^4,23^ 2001, Asia, Australasia, and Europe	Stroke or TIA	Within 5 y	Perindopril, 4 mg, daily	Placebo	2561	32	65	3.9	Recurrent stroke	144/84	Mean difference between the 2 groups throughout the study	5/3
DUTCH TIA,^17^ 1993, the Netherlands	Nondisabling ischemic stroke or TIA	Within 3 mo	Atenolol, 50 mg, daily	Placebo	1473	36	NA	2.6	Death from vascular causes, nonfatal MI, and nonfatal stroke	158/91	Difference between the 2 groups at first follow-up after randomization (median at 4 mo)	5.8/2.9
RESPECT,^3^ 2019, Japan	Stroke	Within 3 y	Lowering BP to <120/80 mm Hg	Lowering BP to <140/90 mm Hg	1263	31	67	3.9	Recurrent stroke	145/85	Mean difference between the 2 groups throughout the study	6.5/3.3
PATS,^20^ 2009, China	Stroke or TIA	≥4 wk	Indapamide, 2.5 mg, daily	Placebo	5665	28	60	2	Recurrent stroke	154/93	Mean difference between 2 groups after 2 y	6.8/3.3
PODCAST,^22^ 2017, UK	Stroke	Within previous 3-7 mo	Lowering SBP to <125 mm Hg	Lowering SBP to <140 mm Hg	83	23	74	2	ACE-R	147/82	Difference between the 2 groups in first 6 mo of treatment	10.6/5.5
SPS 3,^5^ 2013, North America, Latin America, and Spain	Lacunar infarction	Within 180 d	Lowering SBP to <130 mm Hg	Lowering SBP to 130-149 mm Hg	3020	37	63	3.7	Recurrent stroke	143/79	Difference at the last study visit	11/NA
PROGRESS (combined),^4,23^ 2001, Asia, Australasia, and Europe	Stroke or TIA	Within 5 y	Perindopril, 4 mg, daily plus indapamide, 2.5 mg, daily	Placebo	3544	29	63	3.9	Recurrent stroke	149/87	Mean difference between the 2 groups throughout the study	12/5
Liu et al,^19^ 2005, China	Stroke or TIA	Within 5 y	Perindopril, 4 mg, daily plus indapamide, 2.5 mg, daily	Placebo	1520	29	64	4	Recurrent stroke	145/87	Mean difference between the 2 groups throughout the study	14/6

Abbreviations: ACE-R, Addenbrooke’s Cognitive Examination-Revised; BP, blood pressure; Dutch TIA, Dutch Transient Ischemic Attack; MI, myocardial infarction; NA, not available; PAST-BP, Prevention After Stroke–Blood Pressure; PATS, Post-stroke Antihypertensive Treatment Study; PODCAST, Prevention of Decline in Cognition after Stroke Trial; PRoFESS, Prevention Regimen for Effectively Avoiding Second Strokes; PROGRESS, Perindopril Protection Against Recurrent Stroke Study; RESPECT, Recurrent Stroke Prevention Clinical Outcome; SBP, systolic blood pressure; TEST, Tenormin After Stroke and TIA; TIA, transient ischemic attack.

The definition of differential blood pressure reduction varied among included trials. Among included trials, 5 adopted mean blood pressure difference between the 2 groups throughout the study^2,3,4,19,21^ and 1 adopted blood pressure difference after 1 month of treatment,^18^ 1 adopted difference at first follow-up after randomization (median at 4 months),^17^ 1 adopted mean difference after 2 years,^20^ 1 adopted difference at first 6 months of treatment,^22^ and 1 adopted difference at the last study visit.^5^ The mean SBP reduction was 6.7 mm Hg and the mean DBP reduction was 2.8 mm Hg between active treatment and comparator groups. The Cochrane risk-of-bias assessment for the included trials is summarized in eFigure 2 in Supplement 1.

### Primary Outcome

#### Recurrent Stroke

**Figure 1.**
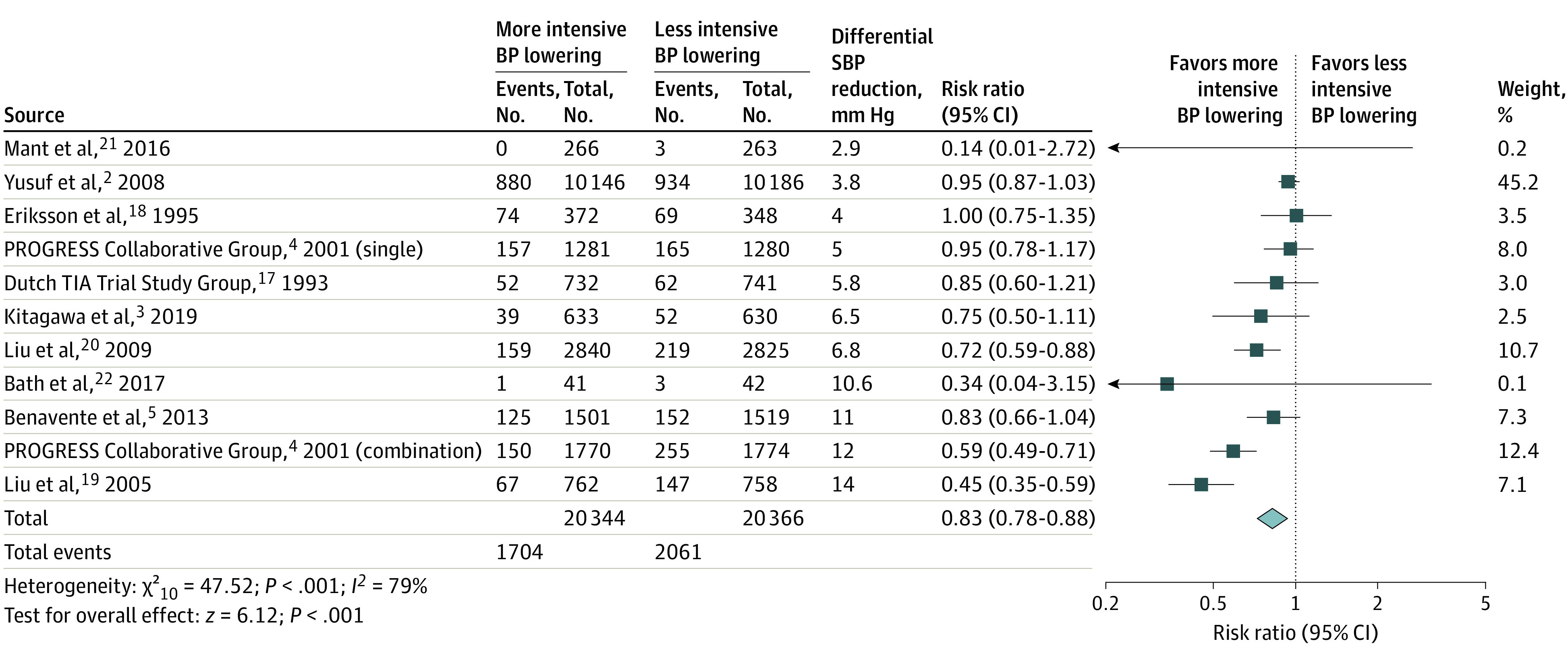
Risk of Recurrent Stroke Risk ratio with 95% CI of recurrent stroke with more intensive compared with less intensive blood pressure (BP)–lowering therapy in patients with stroke or transient ischemic attack (TIA). PROGRESS indicates Perindopril Protection Against Recurrent Stroke Study; SBP, systolic blood pressure.

Pooled results from the fixed-effects model showed that more intensive compared with less intensive blood pressure–lowering therapy was associated with a reduced risk of recurrent stroke in patients with stroke or TIA (10 trials; absolute risk, 8.4% vs 10.1%; RR, 0.83; 95% CI, 0.78-0.88; *P* < .001; number needed to treat [NNT] in 3 years, 58). There was considerable inconsistency among included trials (*P* for heterogeneity < .001; *I^2^* = 79%) (Figure 1).^2,3,4,5,17,18,19,20,21,22^ Pooled results with the random-effects model obtained similar results.

**Figure 2.**
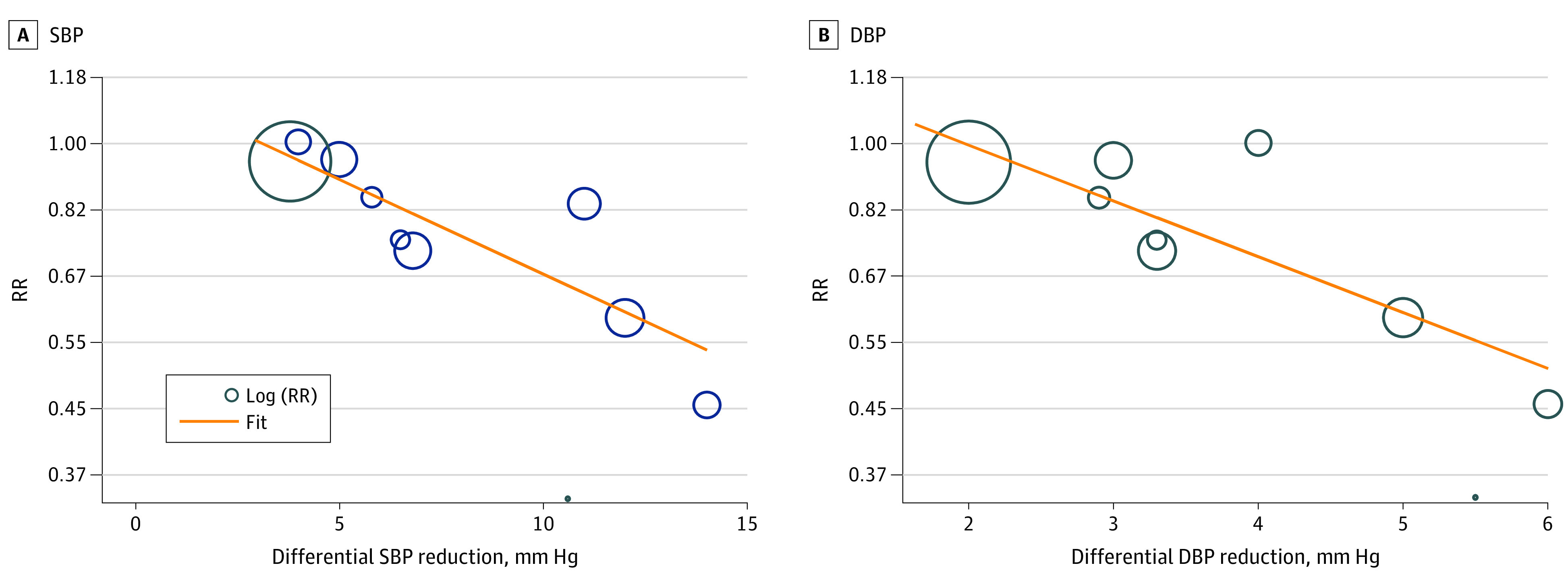
Meta-Regression of Recurrent Stroke Meta-regression of included trials to explore the association between magnitude of differential systolic blood pressure (SBP) reduction (A) and differential diastolic blood pressure (DBP) reduction (B) vs recurrent stroke rate. RR indicates risk ratio.

Meta-regression showed that the magnitude of differential SBP reduction was associated with a lower risk of recurrent stroke in patients with stroke or TIA in a log-linear fashion (regression slope, −0.06; 95% CI, −0.08 to −0.03; *P* = .001) (Figure 2A).^2,3,4,5,17,18,19,20,21,22^ A 5-mm Hg greater SBP reduction was associated with an RR of 0.90 and a 10-mm Hg greater SBP reduction with an RR of 0.67. Also, differential magnitude of DBP reduction was associated with a lower risk of recurrent stroke in patients with stroke or TIA in a log-linear fashion (regression slope, −0.17; 95% CI, −0.26 to −0.08; *P* = .003) (Figure 2B).^2,3,4,17,18,19,20,21,22^ A 3-mm Hg greater DBP reduction was associated with an RR of 0.84 and a 5-mm Hg greater DBP reduction with an RR of 0.60.

With regard to subgroups of trials with different magnitudes of differential SBP reduction, pooled results showed that more intensive compared with less intensive blood pressure–lowering therapy was associated with a lower effect magnitude estimand when the amount of differential SBP reduction was 4 mm Hg or lower (3 trials; RR, 0.95; 95% CI, 0.87-1.03)^2,18,21^ or 5 mm Hg or lower (4 trials; RR, 0.95; 95% CI, 0.88-1.02)^2,4,18,21^ and associated with a greater effect magnitude when the amount of differential SBP reduction was more than 5 mm Hg (7 trials; RR, 0.67; 95% CI, 0.60-0.74; NNT = 28),^3,4,5,17,19,20,22^ more than 7 mm Hg (4 trials; RR, 0.62; 95% CI, 0.54-0.70; NNT = 19),^4,5,19,22^ and more than 11 mm Hg (2 trials; RR, 0.54; 95% CI, 0.46-0.63; NNT = 14) (Table 2).^4,19^

**Table 2.** Association Between Different Reduction Magnitude of SBP and DBP and Recurrent Stroke Risk

Differential BP reduction magnitude	No. of events/No. of population (%)		RR (95% CI)	NNT in 3 y
	More intensive	Less intensive		
**Differential SBP reduction magnitude**				
≤4 mm Hg^2,18,21^	954/10 784 (8.8)	1006/10 797 (9.3)	0.95 (0.87-1.03)	NA
≤5 mm Hg^2,4,18,21^	1111/12 065 (9.2)	1171/12 077 (9.7)	0.95 (0.88-1.02)	NA
>5 mm Hg^3,4,5,17,19,20,22^	593/8279 (7.2)	890/8289 (10.7)	0.67 (0.60-0.74)	28 (23-36)
>7 mm Hg^4,5,19,22^	343/4074 (8.4)	557/4093 (13.6)	0.62 (0.54-0.70)	19 (16-25)
>11 mm Hg^4,19^	217/2532 (8.5)	402/2532 (15.9)	0.54 (0.46-0.63)	14 (12-17)
**Differential DBP reduction magnitude**				
≤2 mm Hg^2,21^	880/10 412 (8.5)	937/10 499 (8.9)	0.94 (0.86-1.03)	NA
≤3 mm Hg^2,4,17,18,21^	1163/12 797 (9.1)	1233/12 818 (9.6)	0.94 (0.87-1.02)	NA
>3 mm Hg^3,4,19,20,22^	416/6046 (6.9)	676/6029 (11.2)	0.61 (0.55-0.69)	23 (20-29)
>4 mm Hg^4,19,22^	218/2573 (8.5)	405/2574 (15.7)	0.54 (0.46-0.63)	14 (12-17)

Abbreviations: BP, blood pressure; DBP, diastolic blood pressure; NA, not applicable; NNT, number needed to treat; RR, risk ratio; SBP, systolic blood pressure.

With regard to subgroups of trials with different magnitudes of differential DBP reduction, pooled results showed that more intensive compared with less intensive blood pressure–lowering therapy was associated with a lower effect magnitude estimand when the amount of differential DBP reduction was 2 mm Hg or lower (2 trials; RR, 0.94; 95% CI, 0.86-1.03)^2,21^ or 3 mm Hg or lower (5 trials; RR, 0.94; 95% CI, 0.87-1.02)^2,4,17,18,21^ and associated with a greater effect magnitude when the amount of differential DBP reduction was more than 3 mm Hg (5 trials; RR, 0.61; 95% CI, 0.55-0.69; NNT = 23)^3,4,19,20,22^ and more than 4 mm Hg (3 trials; RR, 0.54; 95% CI, 0.46-0.63; NNT = 14) (Table 2).^4,19,22^

### Secondary Outcomes

#### Major Cardiovascular Events

Pooled results showed that more intensive compared with less intensive blood pressure–lowering therapy was associated with a reduced risk of major cardiovascular events in patients with stroke or TIA (9 trials; absolute risk, 12.0% vs 13.7%; RR, 0.88; 95% CI, 0.83-0.92; *P* < .001; NNT in 3 years, 61). There was substantial heterogeneity among included trials (*P* for heterogeneity < .001; *I^2^* = 71%) (eFigure 3 in Supplement 1).^2,3,4,5,17,18,19,20,21,22^

**Figure 3.**
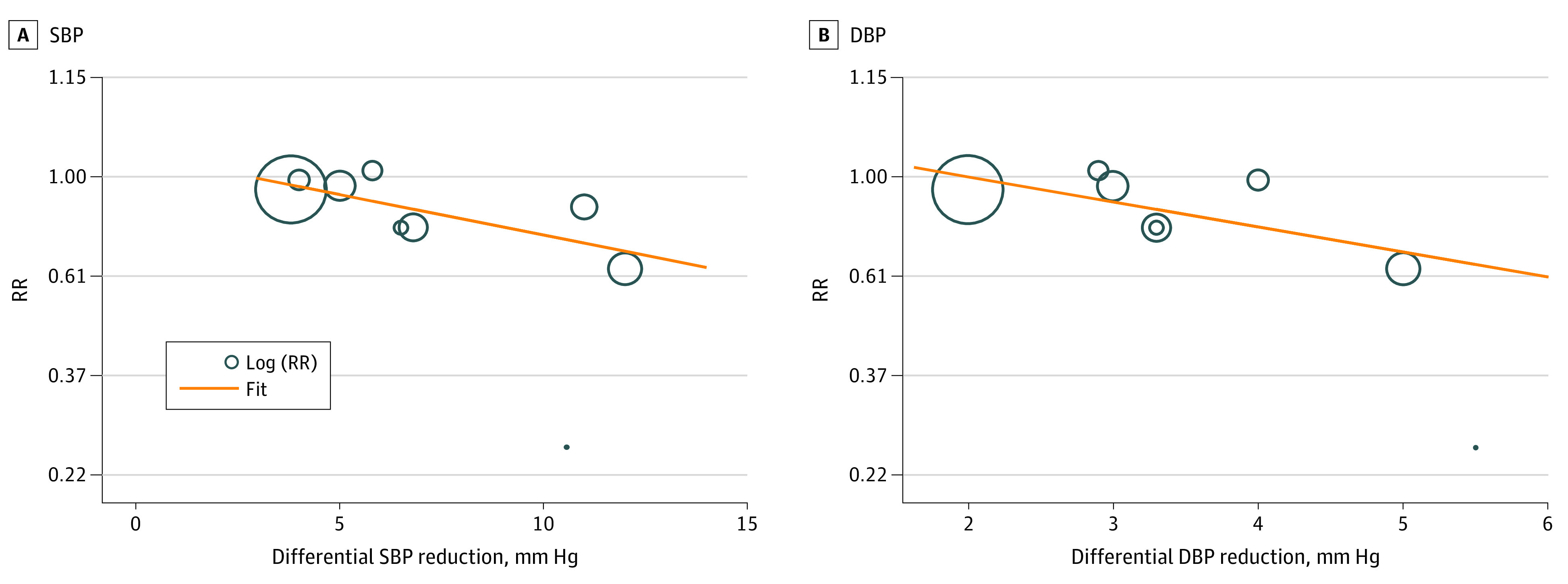
Meta-Regression of Major Cardiovascular Events Meta-regression of included trials to explore the association between magnitude of (A) differential systolic blood pressure (SBP) reduction and (B) differential diastolic blood pressure (DBP) reduction vs major cardiovascular events. RR indicates risk ratio.

Meta-regression showed that the magnitude of differential SBP reduction was associated with a lower risk of major cardiovascular events in patients with stroke or TIA in a log-linear fashion (regression slope, −0.04; 95% CI, −0.07 to −0.01; *P* = .01) (Figure 3A).^2,3,4,5,17,18,19,21,22^ A 5-mm Hg greater SBP reduction was associated with an RR of 0.92, and a 10-mm Hg greater SBP reduction with an RR of 0.75. Also, magnitude of differential DBP reduction was associated with a lower risk of major cardiovascular events in patients with stroke or TIA in a log-linear fashion (regression slope, −0.12; 95% CI, −0.25 to 0; *P* = .048) (Figure 3B).^2,3,4,17,18,20,21,22^ A 3-mm Hg greater DBP reduction was associated with an RR of 0.88 and a 5-mm Hg greater DBP reduction with an RR of 0.69.

#### Ischemic Stroke, Hemorrhagic Stroke, and Fatal or Disabling Stroke

Pooled results showed that more intensive compared with less intensive blood pressure–lowering therapy was associated with a reduced risk of recurrent ischemic stroke in patients with stroke or TIA (6 trials; absolute risk, 7.5% vs 8.7%; RR, 0.87; 95% CI, 0.81-0.94; *P* < .001; NNT = 88). There was considerable inconsistency among included trials (*P* for heterogeneity = .001; *I^2^* = 75%) (eFigure 4 in Supplement 1).^2,3,4,5,19,22^

Pooled results showed that more intensive compared with less intensive blood pressure–lowering therapy was associated with a reduced risk of hemorrhagic stroke in patients with stroke or TIA (6 trials; absolute risk, 0.7% vs 1.3%; RR, 0.54; 95% CI, 0.43-0.68; *P* < .001; NNT = 167). There was substantial heterogeneity among included trials (*P* for heterogeneity = .007; *I^2^* = 69%) (eFigure 5 in Supplement 1).^2,3,4,5,19,22^

Pooled results showed that more intensive compared with less intensive blood pressure–lowering therapy was associated with a reduced risk of fatal or disabling stroke in patients with stroke or TIA (6 trials; absolute risk, 3.0% vs 3.9%; RR, 0.76; 95% CI, 0.64-0.89; *P* < .001; NNT = 107). Heterogeneity was not important among included trials (*P* for heterogeneity = .52; *I^2^* = 0%) (eFigure 6 in Supplement 1).^4,5,17,18,20,22^

#### Myocardial Infarction

Pooled results showed that more intensive compared with less intensive blood pressure–lowering therapy was not associated with a significantly reduced risk of myocardial infarction in patients with stroke or TIA (10 trials; absolute risk, 1.8% vs 2.0%; RR, 0.89; 95% CI, 0.78-1.03; *P* = .11). Heterogeneity was not important among included trials (*P* for heterogeneity = .31; *I^2^* = 15%) (eFigure 7 in Supplement 1).^2,3,4,5,17,18,19,20,21,22^

#### Death From Cardiovascular Causes

Pooled results showed that more intensive compared with less intensive blood pressure–lowering therapy was associated with a reduced risk of death from cardiovascular causes in patients with stroke or TIA (9 trials; absolute risk, 3.2% vs 3.7%; RR, 0.86; 95% CI, 0.78-0.96; *P* = .006; NNT = 193). Heterogeneity was not important among included trials (*P* for heterogeneity = .46; *I^2^* = 0%) (eFigure 8 in Supplement 1).^2,4,5,17,18,19,20,21,22^

#### Death From Any Cause

Pooled results showed that more intensive compared with less intensive blood pressure–lowering therapy was not associated with a significantly reduced risk of death from any cause in patients with stroke or TIA (10 trials; absolute risk, 7.4% vs 7.6%; RR, 0.97; 95% CI, 0.91-1.04; *P* = .42). Heterogeneity was not important among included trials (*P* for heterogeneity = .31; *I^2^* = 14%) (eFigure 9 in Supplement 1).^2,3,4,5,17,18,19,20,21,22^

#### Heart Failure

Pooled results showed that more intensive compared with less intensive blood pressure–lowering therapy was not associated with a reduced risk of heart failure in patients with stroke or TIA (2 trials; absolute risk, 1.2% vs 1.1%; RR, 1.05; 95% CI, 0.82-1.35; *P* = .68). Heterogeneity was not important among included trials (*P* for heterogeneity = .53; *I^2^* = 0%) (eFigure 10 in Supplement 1).^2,3^

Summaries of evidence for the primary and secondary outcomes are presented in the eTable in Supplement 1.

#### Sensitivity Tests

Sensitivity testing removing each individual trial from the meta-analysis one at a time yielded pooled results similar to the overall pooled estimates of the primary outcome. Sensitivity testing restricting analysis to trials with recurrent stroke being the primary outcome in the original trial design showed that more intensive treatment compared with less intensive or no treatment was associated with a reduced risk of recurrent stroke in patients with stroke or TIA (6 trials; RR, 0.82; 95% CI, 0.77-0.88; *P* < .001) (eFigure 11 in Supplement 1).^2,3,4,5,19,20,22^

#### Subgroup Analysis

Subgroup analyses of included trials showed that more intensive compared with less intensive blood pressure–lowering therapy was associated with a greater risk reduction of recurrent stroke in (1) trials with patients enrolled within 3 to 5 years from stroke (RR, 0.67; 95% CI, 0.59-0.75) than patients enrolled within 6 months from stroke (RR, 0.93; 95% CI, 0.86-1.00; *P* for interaction < .001; *I^2^* = 95%) (eFigure 12 in Supplement 1); (2) trials with study follow-up of 3 years or more (RR, 0.75; 95% CI, 0.68-0.83) than in trials with study duration less than 3 years (RR, 0.88; 95% CI 0.81-0.95; *P* for interaction = .01; *I^2^* = 85%) (eFigure 13 in Supplement 1); (3) trials enrolling an Asian population (RR, 0.63; 95% CI, 0.54-0.73) than in trials enrolling a mostly non-Asian population (RR, 0.88; 95% CI 0.82-0.94; *P* for interaction < .001; *I^2^* = 94%) (eFigure 14 in Supplement 1); and (4) trials using an ACE inhibitor plus diuretics (RR, 0.54; 95% CI, 0.46-0.63) than in trials using either ACE inhibitors, angiotensin receptor blockers, β-blockers, diuretics alone (*P* for interaction < .001; *I^2^* = 91%) (eFigure 15 in Supplement 1). In contrast, no significant heterogeneity was found between trials with (1) mean baseline SBP of 140 to 149 mm Hg vs 150 mm Hg or higher; (2) achieved SBP in intensive treated group of less than 130 mm Hg vs 130 to 139 mm Hg vs 140 mm Hg or higher; (3) achieved SBP in less intensive or no therapy of less than 130 mm Hg vs 130 to 139 mm Hg vs 140 mm Hg or higher; (4) entry event ischemic vs hemorrhagic stroke; (5) trial sample size less than 3000 vs 3000 or more; (6) study design (antihypertensive drugs vs placebo and a lower blood pressure target vs a higher blood pressure target); and (7) definition of differential blood pressure reduction (mean difference throughout the studies vs other definitions) (eFigures 16-22 in Supplement 1).

#### Publication Bias

There was no obvious publication bias assessed by the trim-and-fill method for the primary outcome (eFigure 23 in Supplement 1).

## Discussion

The present meta-analysis, comprising 10 randomized clinical trials for blood pressure–lowering therapy enrolling over 40 000 individuals with a history of stroke or TIA, revealed that more intensive compared with less intensive blood pressure–lowering therapy was associated with a risk reduction of recurrent stroke. Further, meta-regression suggested the magnitudes of both differential SBP and DBP reduction were monotonically associated with a lower risk of recurrent stroke. More intensive compared with less intensive blood pressure–lowering therapy was also associated with a risk reduction of major cardiovascular events, ischemic stroke, hemorrhagic stroke, fatal or disabling stroke, and death from cardiovascular causes in patients with stroke or TIA.

High statistical heterogeneity as well as clinical heterogeneity was found in the forest plots in the primary outcome (ie, recurrent stroke) and the lead secondary outcome (ie, major cardiovascular events). In the forest plot of the recurrent stroke, the larger of the differential SBP reduction, the larger of the reduction of recurrent stroke was found visually. In the meta-regression analyses, magnitude of differential SBP and DBP reduction were significantly associated with a lower risk of recurrent stroke in patients with stroke or TIA. Therefore, heterogeneity might be primarily driven by the various magnitude of differential blood pressure lowering among included trials. Similar findings were noted in the major cardiovascular events.

The current study was distinct from the study by Katsanos et al^8^ in several aspects. First, we excluded trials comparing one antihypertensive drug vs another antihypertensive drug (eg, eprosartan vs nitrendipine)^24^ or achieved SBP higher in more intensive blood pressure–lowering group than less intensive blood pressure–lowering group after treatment.^25^ This approach was taken to be in line with the objective of our study. Second, the study by Katsanos et al^8^ only pooled data from trials with antihypertensive therapy vs placebo, whereas we pooled data from trials with antihypertensive therapy vs placebo and a lower blood pressure target vs a higher blood pressure target. The latter approach includes all relevant trials of blood pressure–lowering therapy for secondary stroke prevention. Third, the study by Katsanos et al^8^ adopted achieved blood pressure levels for meta-regression analyses, whereas this study adopted magnitude of differential blood pressure reduction for meta-regression analyses and showed that the larger the magnitude of differential blood pressure reduction, the larger the reduction of recurrent stroke and major cardiovascular events.

The current study is consonant with and extends prior investigations. In the current study, greater achieved differential blood pressure lowering was beneficial even among patients at the lower end of the blood pressure spectrum. The degree of separation between more intensive and less intensive SBP lowering was associated with a uniform relative risk reduction regardless of whether patients with less intensive treatment SBP levels of 140 mm Hg or higher, 130-139 mm Hg, or less than 130 mm Hg. This finding accords with analyses of intensive blood pressure lowering in stroke qualifying event trials, including PROGRESS and the Warfarin-Aspirin Symptomatic Intracranial Disease (WASID) trial.^26,27^ It accords as well with studies in broader cardiovascular event qualifying event trials, including the Action to Control Cardiovascular Risk in Diabetes Blood Pressure (ACCORD BP) trial,^28^ the Systolic Blood Pressure Intervention Trial (SPRINT),^29,30^ and a trial of older Asian individuals with hypertension.^31^ Furthermore, physiologically, intensive (SBP target <120 mm Hg) compared with standard (SBP target <140 mm Hg) blood pressure–lowering therapy was associated with increased, not decreased, cerebral perfusion, in participants with a history of cardiovascular disease.^32^ Taken together, this study does not refute the current practice guidelines recommending blood pressure lowering to an SBP target of less than 130 mm Hg as a major target for patients with stroke or TIA.

Among patients with high-grade vertebrobasilar or other intracranial stenoses, an inverse association of SBP with recurrent stroke risk was found in some studies.^33,34^ However, post hoc analyses of several trials showed that treating SBP to a target of less than 140 mm Hg in patients with severe intracranial stenosis might be beneficial.^27,35,36,37^ Since the risk-benefit profile of more aggressive blood pressure lowering is not consistent among studies, secondary prevention guidelines recommend a less aggressive SBP target (<140 mm Hg) in patients with 50% to 99% stenosis of a major intracranial artery.^38^

Although this meta-analysis excluded trials with blood pressure–lowering therapy used in acute stage of stroke or TIA, the time interval from stroke to randomization still varied among included trials. A subgroup analysis suggested that more intensive compared with less intensive blood pressure–lowering therapy was associated with substantial risk reduction of recurrent stroke in trials with patients enrolled within 3 to 5 years from stroke or TIA but only associated with modest risk reduction of recurrent stroke in trials with patients enrolled within 6 months from stroke or TIA. This finding accords with observational studies that have suggested that aggressive blood pressure lowering might not be beneficial within the first 6 months following the index ischemic stroke event.^39,40^ These findings support a strategy of a moderate blood pressure–lowering target during the first 6 months following stroke or TIA followed by a more aggressive target chronically.

A subgroup analysis also suggested that more intensive compared with less intensive blood pressure–lowering therapy was associated with a larger risk reduction of recurrent stroke in trials with study duration of 3 years or more vs less than 3 years. It is conceivable that a longer duration of high blood pressure levels may cause more profound damage of vessels thereby leading to a higher likelihood of recurrent stroke in patients with stroke or TIA. Also, we found that more intensive compared with less intensive blood pressure–lowering therapy was associated with a larger risk reduction of recurrent stroke in Asian populations than non-Asian populations. In general, the ratio of hemorrhagic stroke to ischemic stroke is higher in Asian than in non-Asian populations,^41^ and this meta-analysis showed that more intensive compared with less intensive blood pressure–lowering therapy was associated with a substantial relative risk reduction in hemorrhagic stroke (RR, 0.54; 95% CI, 0.43-0.68). Also, the risk of recurrent ischemic and hemorrhagic stroke was greater in Asian population with high blood pressure compared with several other groups around the world.^16^ Therefore, it is conceivable that more intensive blood pressure–lowering treatment might be especially beneficial for Asian populations.

### Limitations

There are several limitations to this study. First, although it was conceivable that some patients in the included trials had baseline SBP less than 140 mm Hg, none of included trials had mean baseline SBP less than 140 mm Hg. Granular analysis related to baseline BP levels could not be conducted because this study was a trial-level meta-analysis, rather than an individual, patient-level pooled analysis. Second, the purpose of some included trials was not to evaluate association between blood pressure lowering and risk of recurrent stroke,^17,18,21,22^ and in such settings, there might be a higher chance that stroke events were not well recorded. Still, a sensitivity test restricting analysis within trials with recurrent stroke reported as the primary outcome obtained similar results. Third, although we excluded trials before 1980, the included trials reflect a long period of clinical practice during which concomitant treatments for stroke prevention further evolved.^1^ However, the consistency in benefit of blood pressure lowering suggests substantial benefit on top of a wide range of background therapies. Fourth, the individual studies mostly have wide CIs and many of the pooled results of secondary outcomes are driven by a single study with a large weight. Fifth, more than half of the participants are contributed by the Prevention Regimen for Effectively Avoiding Second Strokes (PRoFESS)^2^ and PROGRESS^4^ trials. Still, sensitivity testing removing either trial yielded pooled results similar to the overall pooled estimates of the primary outcome.

## Conclusions

This meta-analysis and meta-regression of randomized clinical trials for blood pressure–lowering therapy found that more intensive compared with less intensive blood pressure–lowering therapy may be associated with a reduction of recurrent stroke; further, the larger the magnitude of differential blood pressure reduction, the larger the reduction of cerebrovascular events. More intensive blood pressure–lowering therapy may be also associated with a risk reduction of major cardiovascular events, ischemic stroke, hemorrhagic stroke, fatal or disabling stroke, and death from cardiovascular causes in patients with stroke or TIA. These results might support the use of more intensive blood pressure reduction for secondary stroke prevention chronically.

## Notes

Supplement 1. eFigure 1. Study selection eFigure 2. Risk of bias eFigure 3. Major cardiovascular events eFigure 4. Recurrent ischemic stroke eFigure 5. Hemorrhagic stroke eFigure 6. Fatal or disabling stroke eFigure 7. Myocardial infarction eFigure 8. Death from cardiovascular causes eFigure 9. Death from any cause eFigure 10. Heart failure eFigure 11. Sensitivity testing eFigure 12. Time interval from index stroke to randomization eFigure 13. Study duration eFigure 14. Ethnicity eFigure 15. Antihypertensive drugs used in more intensive treated group eFigure 16. Mean baseline SBP eFigure 17. Achieved SBP in more intensive treated group eFigure 18. Achieved SBP in less intensive treated group eFigure 19. Entry event eFigure 20. Sample size eFigure 21. Study design eFigure 22. Definition of differential blood pressure reduction eFigure 23. Publication bias eTable. Grading of Recommendations, Assessment, Development and Evaluations (GRADE) eReferences

Supplement 2. Data sharing statement
