# Supplementary material for: Association Between Magnitude of Differential Blood Pressure Reduction and Secondary Stroke Prevention: A Meta-analysis and Meta-Regression
Source: JAMA Neurol. 2023 Mar 20;80(5):506–15. doi: 10.1001/jamaneurol.2023.0218 (PMC10028545; doi:10.1001/jamaneurol.2023.0218)
Supplement: Supplement 1. — eFigure 1. Study selection eFigure 2. Risk of bias eFigure 3. Major cardiovascular events eFigure 4. Recurrent ischemic stroke eFigure 5. Hemorrhagic stroke eFigure 6. Fatal or disabling stroke eFigure 7. Myocardial infarction eFigure 8. Death from cardiovascular causes eFigure 9. Death from any cause eFigure 10. Heart failure eFigure 11. Sensitivity testing eFigure 12. Time interval from index stroke to randomization eFigure 13. Study duration eFigure 14. Ethnicity eFigure 15. Antihypertensive drugs used in more intensive treated group eFigure 16. Mean baseline SBP eFigure 17. Achieved SBP in more intensive treated group eFigure 18. Achieved SBP in less intensive treated group eFigure 19. Entry event eFigure 20. Sample size eFigure 21. Study design eFigure 22. Definition of differential blood pressure reduction eFigure 23. Publication bias eTable. Grading of Recommendations, Assessment, Development and Evaluations (GRADE) eReferences [file jamaneurol-e230218-s001.pdf]

Supplemental Online Content

Hsu CY, Saver JL, Ovbiagele B, Wu YL, Cheng CY, Lee M. Association between magnitude of differential blood pressure reduction and secondary stroke prevention: a meta-analysis and meta-regression. *JAMA Neurol*. Published online March 20, 2023. doi:10.1001/jamaneurol.2023.0218

eFigure 1. Study selection
eFigure 2. Risk of bias
eFigure 3. Major cardiovascular events
eFigure 4. Recurrent ischemic stroke
eFigure 5. Hemorrhagic stroke
eFigure 6. Fatal or disabling stroke
eFigure 7. Myocardial infarction
eFigure 8. Death from cardiovascular causes
eFigure 9. Death from any cause
eFigure 10. Heart failure
eFigure 11. Sensitivity testing
eFigure 12. Time interval from index stroke to randomization
eFigure 13. Study duration
eFigure 14. Ethnicity
eFigure 15. Antihypertensive drugs used in more intensive treated group
eFigure 16. Mean baseline SBP
eFigure 17. Achieved SBP in more intensive treated group
eFigure 18. Achieved SBP in less intensive treated group
eFigure 19. Entry event
eFigure 20. Sample size
eFigure 21. Study design
eFigure 22. Definition of differential blood pressure reduction
eFigure 23. Publication bias
eTable. Grading of Recommendations, Assessment, Development and Evaluations (GRADE)
eReferences

This supplemental material has been provided by the authors to give readers additional information about their work.

© 2023 Hsu CY et al. *JAMA Neurology.*

eTable. Grading of Recommendations, Assessment, Development and Evaluations (GRADE)

Legends: Summary of the quality assessments and summary finding for the primary and secondary outcomes

Quality assessment							Summary of findings				
							Event, No./Total, No.		Effect		Quality
Outcomes, No. of studies	Design	Limitations	Inconsistency	Indirectness	Imprecision	Publication bias	More intensive	Less intensive	Relative (95% CI)	Absolute	
Recurrent stroke, n=10	RCT	No serious limitations	some inconsistency	No serious indirectness	No serious imprecision	Undetected	1704/20344	2061/20366	0.83 (0.78-0.88)	17 fewer per 1000 (12-22)	High
Major cardiovascular events, n=9	RCT	No serious limitations	some inconsistency	No serious indirectness	No serious imprecision	Undetected	2348/19582	2679/19608	0.88 (0.83-0.92)	16 fewer per 1000 (11-23)	High
Ischemic stroke, n=6	RCT	No serious limitations	some inconsistency	No serious indirectness	some imprecision	Undetected	1217/16134	1403/16189	0.87 (0.81-0.94)	11 fewer per 1000 (5-17)	Moderate
Hemorrhagic stroke, n=6	RCT	No serious limitations	some inconsistency	No serious indirectness	some imprecision	Undetected	114/16134	212/16189	0.54 (0.43-	6 fewer per 1000 (4-7)	Moderate

© 2023 Hsu CY et al. *JAMA Neurology.*

									0.68)		
Fatal or disabling stroke, n=6	RCT	No serious limitations	some inconsistency	No serious indirectness	some imprecision	Undetected	253/8537	334/8539	0.76 (0.64-0.89)	9 fewer per 1000 (4-14)	Moderate
Myocardial infarction, n=9	RCT	No serious limitations	Serious inconsistency	No serious indirectness	Serious imprecision	Undetected	365/20344	409/20366	0.89 (0.78-1.03)	Not significant	Moderate
Death from cardiovascular causes, n=9	RCT	No serious limitations	No serious inconsistency	No serious indirectness	some imprecision	Undetected	629/19711	728/19736	0.86 (0.78-0.96)	5 fewer per 1000 (1-8)	Moderate
Death from any cause, n=10	RCT	No serious limitations	Some inconsistency	No serious indirectness	some imprecision	Undetected	1511/20344	1554/20366	0.97 (0.91-1.04)	Not significant	Moderate
Heart failure, n=2	RCT	No serious limitations	Serious inconsistency	No serious indirectness	Serious imprecision	Undetected	126/10779	120/10816	1.05 (0.82-1.35)	Not significant	Low

RCT: randomized clinical trial

© 2023 Hsu CY et al. *JAMA Neurology.*

© 2023 Hsu CY et al. *JAMA Neurology.*

eFigure 1. Study selection

Legends: Flow of study selection.

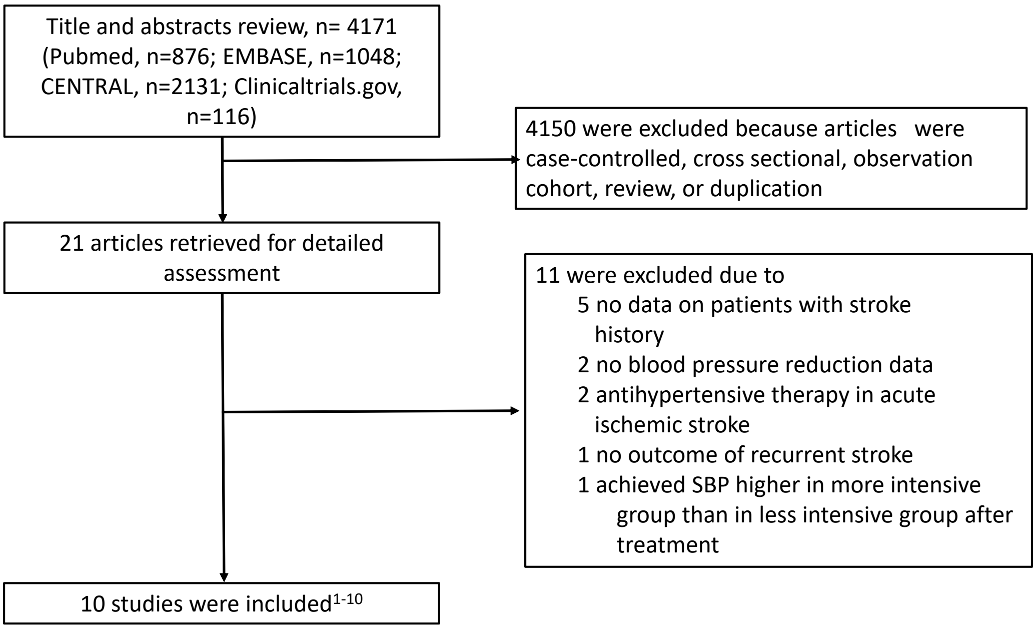

© 2023 Hsu CY et al. *JAMA Neurology.*

eFigure 2. Risk of bias

Legends: Risk of bias for included trials.

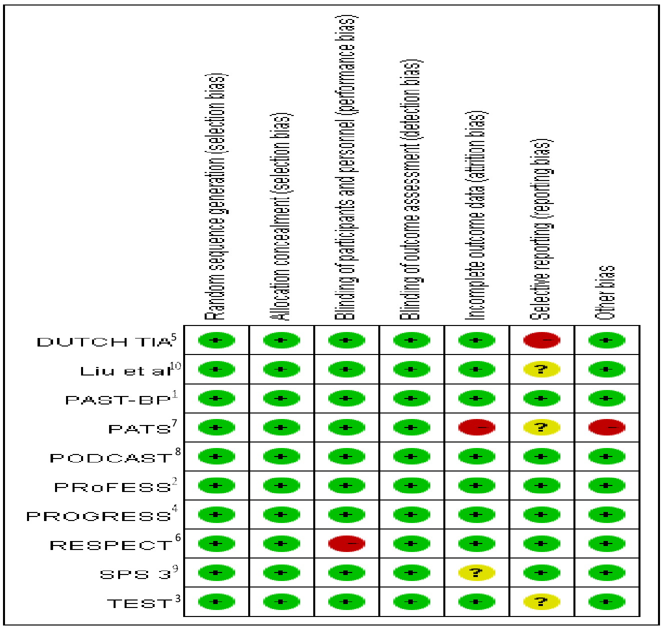

© 2023 Hsu CY et al. *JAMA Neurology.*

eFigure 3: Major cardiovascular events

Legends: Relative risk with 95% confidence interval of major cardiovascular events in more intensive vs less intensive blood pressure lowering in patients with stroke or transient ischemic attack.

	More intensive		Less intensive			Risk Ratio
Study or Subgroup	Events	Total	Events	Total	Weight	M-H, Fixed, 95% CI
PAST-BP [1]	1	266	5	263	0.2%	0.20 [0.02, 1.68]
PRoFESS [2]	1289	10146	1377	10186	51.3%	0.94 [0.88, 1.01]
TEST [3]	97	372	92	348	3.5%	0.99 [0.77, 1.26]
PROGRESS, single [4]	227	1281	237	1280	8.9%	0.96 [0.81, 1.13]
DUTCH TIA [5]	97	732	95	741	3.5%	1.03 [0.79, 1.35]
RESPECT [6]	46	633	59	630	2.2%	0.78 [0.54, 1.12]
PATS [7]	199	2840	255	2825	9.5%	0.78 [0.65, 0.93]
PODCAST [8]	1	41	4	42	0.1%	0.26 [0.03, 2.20]
SPS 3 [9]	160	1501	188	1519	7.0%	0.86 [0.71, 1.05]
PROGRESS, combination [4]	231	1770	367	1774	13.7%	0.63 [0.54, 0.73]
**Total (95% CI)**		**19582**		**19608**	**100.0%**	**0.88 [0.83, 0.92]**
Total events	2348		2679			

Heterogeneity: Chi² = 30.82, df = 9 (P = 0.0003); I² = 71%
Test for overall effect: Z = 5.00 (P < 0.00001)

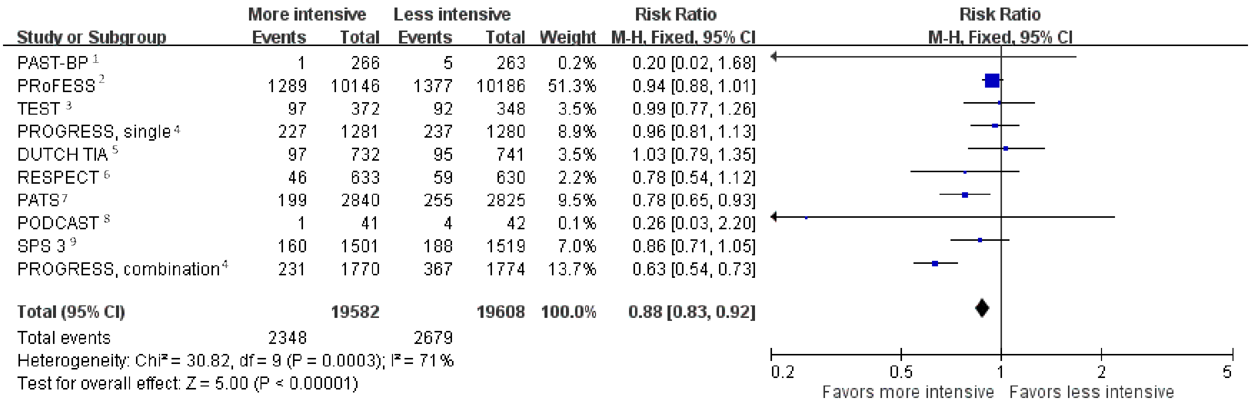

© 2023 Hsu CY et al. *JAMA Neurology.*

eFigure 4. Recurrent ischemic stroke

Legends: Relative risk with 95% confidence interval of recurrent ischemic stroke in more intensive vs less intensive blood pressure lowering in patients with stroke or transient ischemic attack.

	More intensive		Less intensive			Risk Ratio
Study or Subgroup	Events	Total	Events	Total	Weight	M-H, Fixed, 95% CI
PRoFESS[2]	774	10146	811	10186	57.8%	0.96 [0.87, 1.05]
RESPECT [6]	38	633	41	630	2.9%	0.92 [0.60, 1.41]
PROGRESS [11]	246	3051	319	3054	22.8%	0.77 [0.66, 0.90]
PODCAST [8]	0	41	2	42	0.2%	0.20 [0.01, 4.14]
SPS 3 [9]	112	1501	131	1519	9.3%	0.87 [0.68, 1.10]
Liu et al[10]	47	762	99	758	7.1%	0.47 [0.34, 0.66]
**Total (95% CI)**		**16134**		**16189**	**100.0%**	**0.87 [0.81, 0.94]**
Total events	1217		1403			

Heterogeneity: Chi² = 20.13, df = 5 (P = 0.001); I² = 75%
Test for overall effect: Z = 3.70 (P = 0.0002)

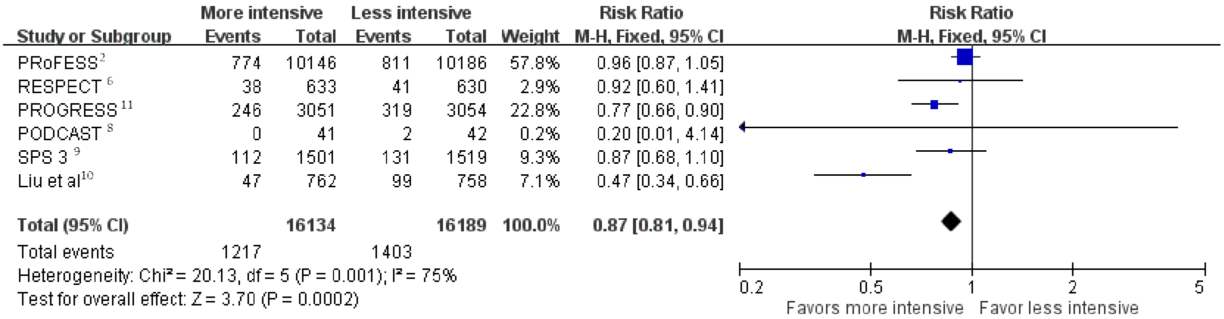

© 2023 Hsu CY et al. *JAMA Neurology.*

eFigure 5. Hemorrhagic stroke

Legends: Relative risk with 95% confidence interval of hemorrhagic stroke in more intensive vs less intensive blood pressure lowering in patients with stroke or transient ischemic attack.

	More intensive		Less intensive			Risk Ratio
**Study or Subgroup**	**Events**	**Total**	**Events**	**Total**	**Weight**	**M-H, Fixed, 95% CI**
PRoFESS[2]	59	10146	69	10186	32.5%	0.86 [0.61, 1.21]
RESPECT[6]	1	633	11	630	5.2%	0.09 [0.01, 0.70]
PROGRESS[11]	37	3051	74	3054	34.9%	0.50 [0.34, 0.74]
PODCAST[8]	1	41	1	42	0.5%	1.02 [0.07, 15.84]
SPS 3[9]	6	1501	16	1519	7.5%	0.38 [0.15, 0.97]
Liu et al[10]	10	762	41	758	19.4%	0.24 [0.12, 0.48]
**Total (95% CI)**		**16134**		**16189**	**100.0%**	**0.54 [0.43, 0.68]**
Total events	114		212			

Heterogeneity: $Chi^2 = 15.98$, $df = 5$ ($P = 0.007$); $I^2 = 69\%$

Test for overall effect: $Z = 5.36$ ($P < 0.00001$)

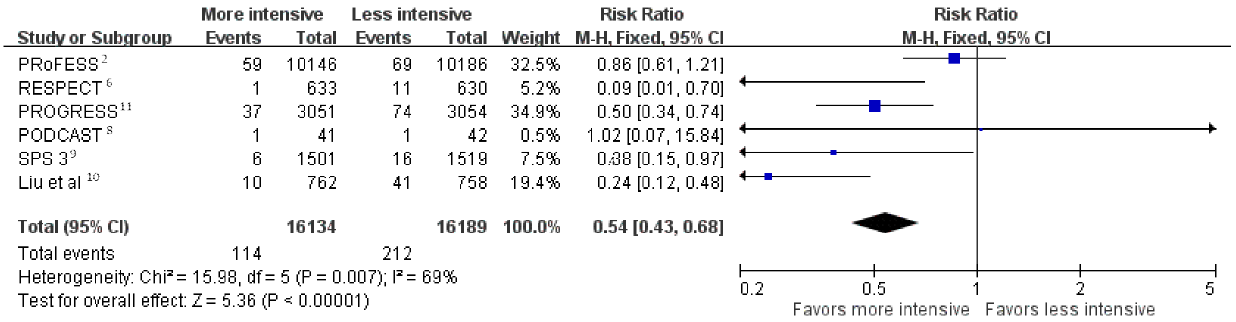

© 2023 Hsu CY et al. *JAMA Neurology.*

eFigure 6. Fatal or disabling stroke

Legends: Relative risk with 95% confidence interval of fatal or disabling stroke in more intensive vs less intensive blood pressure lowering in patients with stroke or transient ischemic attack.

	More intensive		Less intensive			Risk Ratio
Study or Subgroup	Events	Total	Events	Total	Weight	M-H, Fixed, 95% CI
TEST[3]	18	372	17	348	5.2%	0.99 [0.52, 1.89]
DUTCH TIA[5]	11	732	8	741	2.4%	1.39 [0.56, 3.44]
PATS[7]	60	2840	79	2825	23.7%	0.76 [0.54, 1.05]
PROGRESS[4]	123	3051	181	3054	54.0%	0.68 [0.54, 0.85]
PODCAST[8]	1	41	0	42	0.1%	3.07 [0.13, 73.29]
SPS 3[9]	40	1501	49	1519	14.5%	0.83 [0.55, 1.25]
**Total (95% CI)**		**8537**		**8529**	**100.0%**	**0.76 [0.64, 0.89]**
Total events	253		334			

Heterogeneity: Chi² = 4.21, df = 5 (P = 0.52); I² = 0%

Test for overall effect: Z = 3.43 (P = 0.0006)

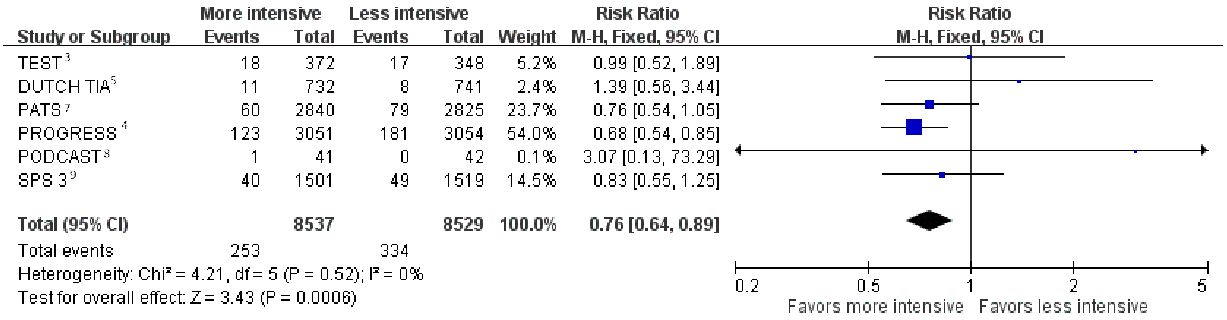

© 2023 Hsu CY et al. *JAMA Neurology.*

eFigure 7. Myocardial infarction

Legends: Relative risk with 95% confidence interval of myocardial infarction in more intensive vs less intensive blood pressure lowering in patients with stroke or transient ischemic attack.

	More intensive		Less intensive			Risk Ratio
Study or Subgroup	Events	Total	Events	Total	Weight	M-H, Fixed, 95% CI
PAST-BP [1]	1	266	1	263	0.2%	0.99 [0.06, 15.72]
PRoFESS [2]	168	10146	169	10186	41.2%	1.00 [0.81, 1.23]
TEST [3]	13	372	14	348	3.5%	0.87 [0.41, 1.82]
DUTCH TIA[5]	45	732	40	741	9.7%	1.14 [0.75, 1.72]
RESPECT [6]	5	633	4	630	1.0%	1.24 [0.34, 4.61]
PATS [7]	26	2840	23	2825	5.6%	1.12 [0.64, 1.97]
PROGRESS [4]	60	3051	96	3054	23.4%	0.63 [0.45, 0.86]
PODCAST [8]	0	41	1	42	0.4%	0.34 [0.01, 8.14]
SPS 3 [9]	36	1501	40	1519	9.7%	0.91 [0.58, 1.42]
Liu et al [10]	11	762	21	758	5.1%	0.52 [0.25, 1.07]
**Total (95% CI)**		**20344**		**20366**	**100.0%**	**0.89 [0.78, 1.03]**
Total events	365		409			

Heterogeneity: Chi² = 10.58, df = 9 (P = 0.31); I² = 15%

Test for overall effect: Z = 1.58 (P = 0.11)

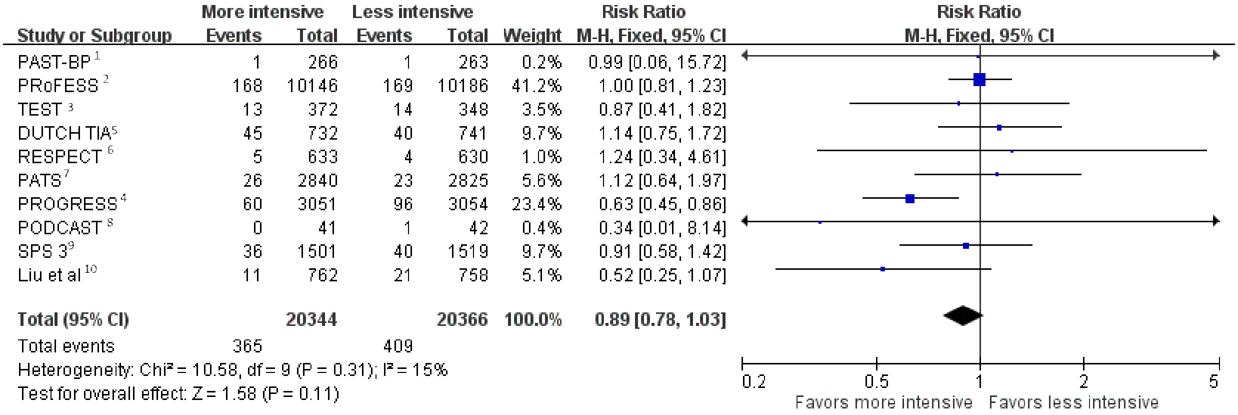

© 2023 Hsu CY et al. *JAMA Neurology.*

eFigure 8. Death from cardiovascular causes

Legends: Relative risk with 95% confidence interval of death from cardiovascular causes in more intensive vs less intensive blood pressure lowering in patients with stroke or transient ischemic attack.

	More intensive		Less intensive			Risk Ratio
Study or Subgroup	Events	Total	Events	Total	Weight	M-H, Fixed, 95% CI
PAST-BP[1]	0	266	1	263	0.2%	0.33 [0.01, 8.05]
PRoFESS[2]	223	10146	263	10186	36.0%	0.85 [0.71, 1.02]
TEST[3]	34	372	39	348	5.5%	0.82 [0.53, 1.26]
DUTCH TIA[5]	41	732	33	741	4.5%	1.26 [0.80, 1.97]
PATS[7]	86	2840	102	2825	14.0%	0.84 [0.63, 1.11]
PROGRESS[4]	181	3051	198	3054	27.1%	0.92 [0.75, 1.11]
PODCAST[8]	1	41	1	42	0.1%	1.02 [0.07, 15.84]
SPS 3[9]	36	1501	41	1519	5.6%	0.89 [0.57, 1.38]
Liu et al[10]	27	762	50	758	6.9%	0.54 [0.34, 0.85]
**Total (95% CI)**		**19711**		**19736**	**100.0%**	**0.86 [0.78, 0.96]**
Total events	629		728			

Heterogeneity: Chi² = 7.71, df = 8 (P = 0.46); I² = 0%

Test for overall effect: Z = 2.77 (P = 0.006)

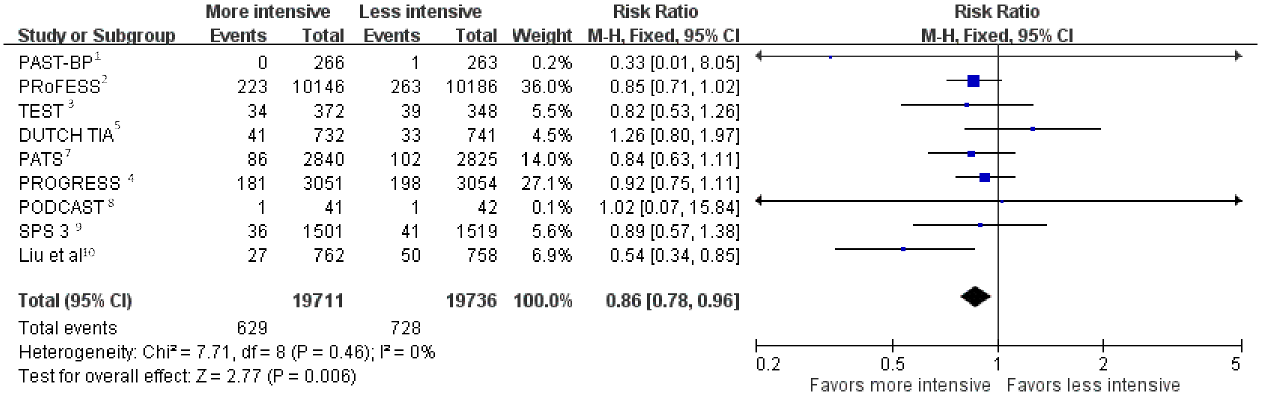

© 2023 Hsu CY et al. *JAMA Neurology.*

eFigure 9. Death from any cause

Legends: Relative risk with 95% confidence interval of death from any cause in more intensive vs less intensive blood pressure lowering in patients with stroke or transient ischemic attack.

	More intensive		Less intensive			Risk Ratio
Study or Subgroup	Events	Total	Events	Total	Weight	M-H, Fixed, 95% CI
PAST-BP[1]	2	266	1	263	0.1%	1.98 [0.18, 21.68]
PRoFESS[2]	755	10146	740	10186	47.5%	1.02 [0.93, 1.13]
TEST[3]	51	372	60	348	4.0%	0.80 [0.56, 1.12]
DUTCH TIA[5]	64	732	58	741	3.7%	1.12 [0.79, 1.57]
RESPECT[6]	30	633	37	630	2.4%	0.81 [0.50, 1.29]
PATS[7]	145	2840	161	2825	10.4%	0.90 [0.72, 1.11]
PROGRESS[4]	306	3051	319	3054	20.5%	0.96 [0.83, 1.11]
PODCAST[8]	4	41	3	42	0.2%	1.37 [0.33, 5.73]
SPS 3[9]	106	1501	101	1519	6.5%	1.06 [0.82, 1.38]
Liu et al[10]	48	762	74	758	4.8%	0.65 [0.46, 0.91]
**Total (95% CI)**		**20344**		**20366**	**100.0%**	**0.97 [0.91, 1.04]**
Total events	1511		1554			

Heterogeneity: Chi² = 10.51, df = 9 (P = 0.31); I² = 14%
Test for overall effect: Z = 0.80 (P = 0.42)

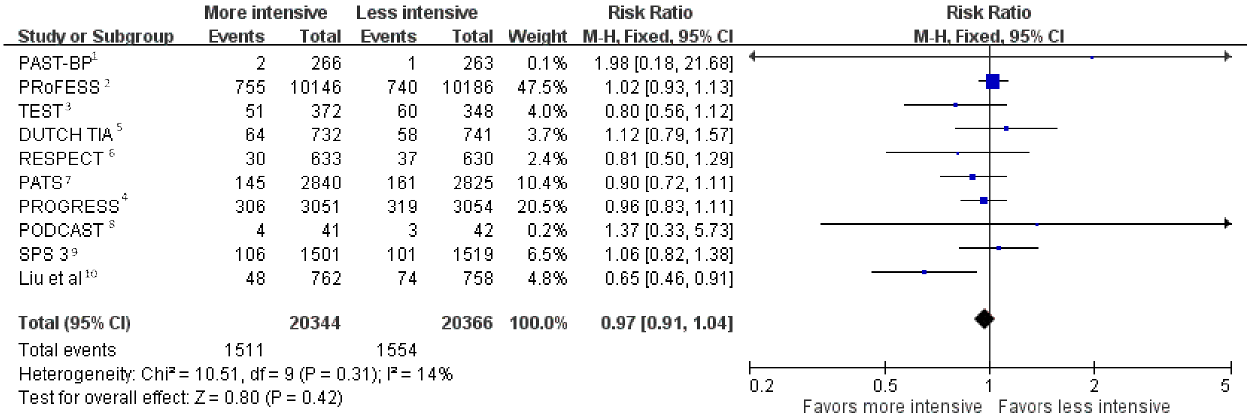

© 2023 Hsu CY et al. *JAMA Neurology.*

eFigure 10. Heart failure

Legends: Relative risk with 95% confidence interval of heart failure in more intensive vs less intensive blood pressure lowering in patients with stroke or transient ischemic attack.

	More intensive		Less intensive			Risk Ratio
Study or Subgroup	Events	Total	Events	Total	Weight	M-H, Fixed, 95% CI
PRoFESS[2]	121	10146	117	10186	97.5%	1.04 [0.81, 1.34]
RESPECT[6]	5	633	3	630	2.5%	1.66 [0.40, 6.91]
**Total (95% CI)**		**10779**		**10816**	**100.0%**	**1.05 [0.82, 1.35]**
Total events	126		120			

Heterogeneity: Chi² = 0.40, df = 1 (P = 0.53); I² = 0%

Test for overall effect: Z = 0.41 (P = 0.68)

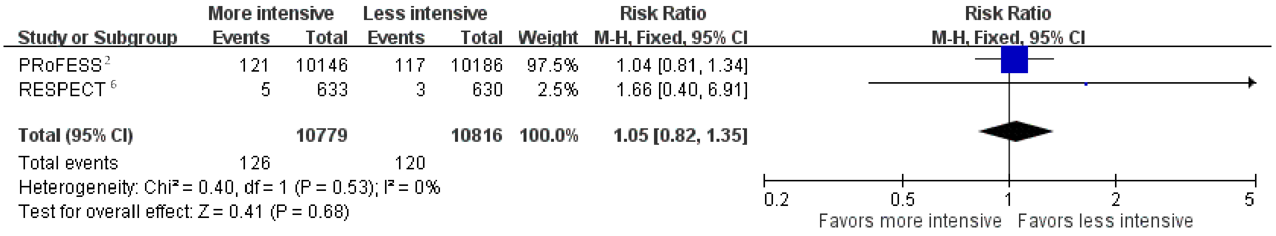

© 2023 Hsu CY et al. *JAMA Neurology.*

eFigure 11. Sensitivity testing

Legends: Relative risk with 95% confidence interval of recurrent stroke in more intensive vs less intensive blood pressure lowering in patients with stroke or transient ischemic attack among trials with recurrent stroke being the primary outcome in the original trial design.

	More intensive		Less intensive			Risk Ratio
Study or Subgroup	Events	Total	Events	Total	Weight	M-H, Fixed, 95% CI
PRoFESS [2]	880	10146	934	10186	48.5%	0.95 [0.87, 1.03]
PROGRESS, single [4]	157	1281	165	1280	8.6%	0.95 [0.78, 1.17]
RESPECT [6]	39	633	52	630	2.7%	0.75 [0.50, 1.11]
PATS [7]	159	2840	219	2825	11.4%	0.72 [0.59, 0.88]
SPS 3 [9]	125	1501	152	1519	7.9%	0.83 [0.66, 1.04]
PROGRESS, combination [4]	150	1770	255	1774	13.3%	0.59 [0.49, 0.71]
Liu et al [10]	67	762	147	758	7.7%	0.45 [0.35, 0.59]
**Total (95% CI)**		**18933**		**18972**	**100.0%**	**0.82 [0.77, 0.88]**
Total events	1577		1924			

Heterogeneity: Chi² = 43.86, df = 6 (P < 0.00001); I² = 86%

Test for overall effect: Z = 6.08 (P < 0.00001)

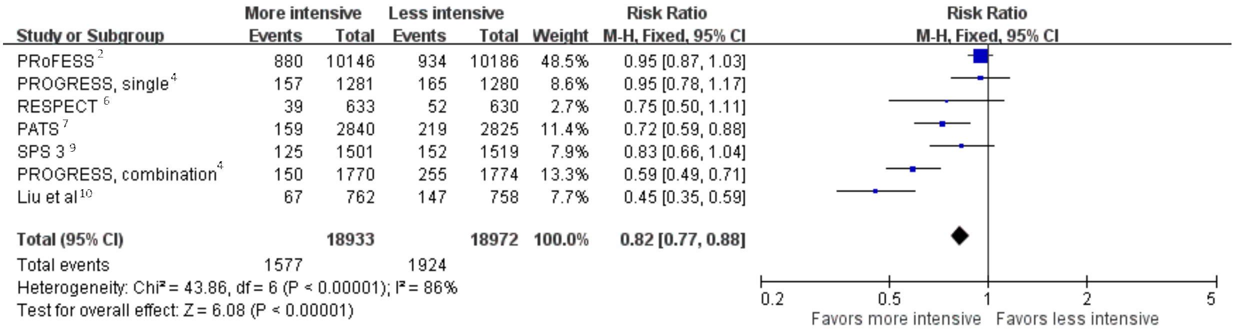

© 2023 Hsu CY et al. *JAMA Neurology.*

eFigure 12. Time interval from index stroke to randomization

Legends: Relative risk with 95% confidence interval of recurrent stroke in more intensive vs less intensive blood pressure lowering in patients with stroke or transient ischemic attack with randomization within 6 months from stroke vs within 3-5 years from stroke.

Study or Subgroup	More intensive Events	More intensive Total	Less intensive Events	Less intensive Total	Weight	Risk Ratio M-H, Fixed, 95% CI
**12.1.1 within 6 months**						
PRoFESS[2]	880	10146	934	10186	76.5%	0.95 [0.87, 1.03]
TEST[3]	74	372	69	348	5.8%	1.00 [0.75, 1.35]
DUTCH TIA[5]	52	732	62	741	5.1%	0.85 [0.60, 1.21]
PODCAST[8]	1	41	3	42	0.2%	0.34 [0.04, 3.15]
SPS 3[9]	125	1501	152	1519	12.4%	0.83 [0.66, 1.04]
**Subtotal (95% CI)**		**12792**		**12836**	**100.0%**	**0.93 [0.86, 1.00]**
Total events	1132		1220			
Heterogeneity: Chi² = 2.37, df = 4 (P = 0.67); I² = 0%						
Test for overall effect: Z = 1.88 (P = 0.06)						
**12.1.2 within 3-5 years**						
PROGRESS, single[4]	157	1281	165	1280	26.7%	0.95 [0.78, 1.17]
RESPECT[6]	39	633	52	630	8.4%	0.75 [0.50, 1.11]
PROGRESS, combination[4]	150	1770	255	1774	41.1%	0.59 [0.49, 0.71]
Liu et al[10]	67	762	147	758	23.8%	0.45 [0.35, 0.59]
**Subtotal (95% CI)**		**4446**		**4442**	**100.0%**	**0.67 [0.59, 0.75]**
Total events	413		619			
Heterogeneity: Chi² = 21.28, df = 3 (P < 0.0001); I² = 86%						
Test for overall effect: Z = 6.77 (P < 0.00001)						

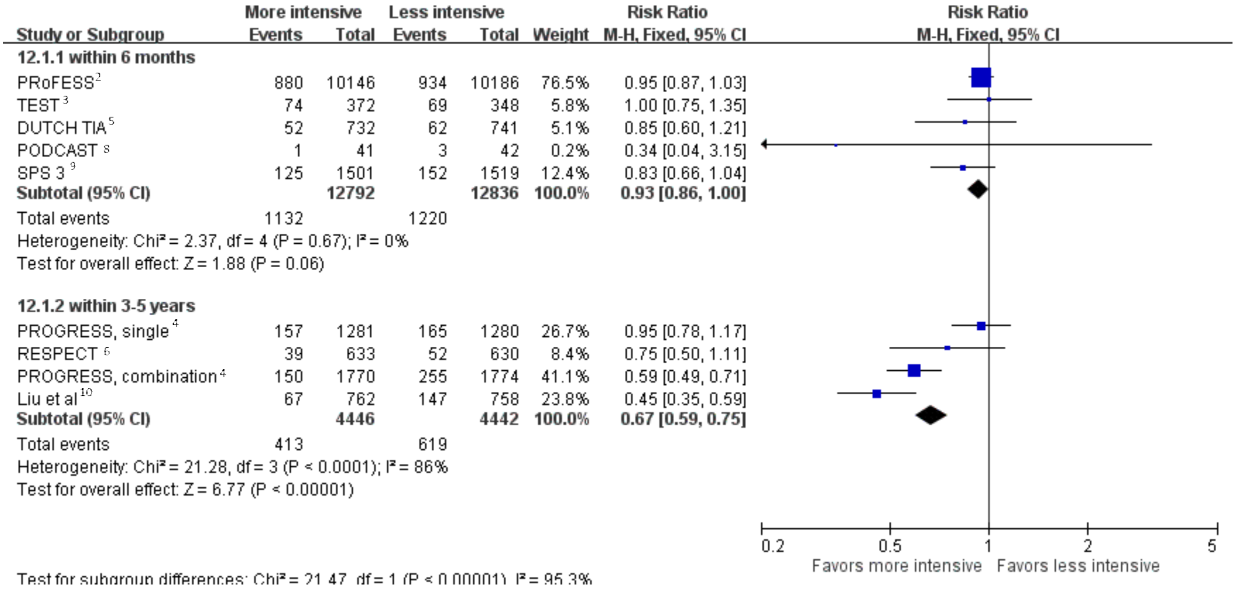

Test for subgroup differences: Chi² = 21.47, df = 1 (P < 0.00001), I² = 95.3%

© 2023 Hsu CY et al. *JAMA Neurology.*

eFigure 13. Study duration

Legends: Relative risk with 95% confidence interval of recurrent stroke in more intensive vs less intensive blood pressure lowering in patients with stroke or transient ischemic attack with study duration < 3 years vs ≥ 3 years.

Study or Subgroup	More intensive Events	More intensive Total	Less intensive Events	Less intensive Total	Weight	Risk Ratio M-H, Fixed, 95% CI
**13.1.1 < 3 years**						
PAST-BP [1]	0	266	3	263	0.3%	0.14 [0.01, 2.72]
PRoFESS [2]	880	10146	934	10186	76.5%	0.95 [0.87, 1.03]
TEST [3]	74	372	69	348	5.8%	1.00 [0.75, 1.35]
DUTCH TIA [5]	52	732	62	741	5.1%	0.85 [0.60, 1.21]
PODCAST [8]	1	41	3	42	0.2%	0.34 [0.04, 3.15]
Liu et al [10]	67	762	147	758	12.1%	0.45 [0.35, 0.59]
**Subtotal (95% CI)**		**12319**		**12338**	**100.0%**	**0.88 [0.81, 0.95]**
Total events	1074		1218			
Heterogeneity: Chi² = 28.59, df = 5 (P < 0.0001); I² = 83%						
Test for overall effect: Z = 3.19 (P = 0.001)						
**13.1.2 ≥ 3 years**						
PROGRESS, single [4]	157	1281	165	1280	19.6%	0.95 [0.78, 1.17]
RESPECT [6]	39	633	52	630	6.2%	0.75 [0.50, 1.11]
PATS [7]	159	2840	219	2825	26.1%	0.72 [0.59, 0.88]
SPS 3 [9]	125	1501	152	1519	17.9%	0.83 [0.66, 1.04]
PROGRESS, combination [4]	150	1770	255	1774	30.2%	0.59 [0.49, 0.71]
**Subtotal (95% CI)**		**8025**		**8028**	**100.0%**	**0.75 [0.68, 0.83]**
Total events	630		843			
Heterogeneity: Chi² = 12.26, df = 4 (P = 0.02); I² = 67%						
Test for overall effect: Z = 5.79 (P < 0.00001)						

Risk Ratio M-H, Fixed, 95% CI

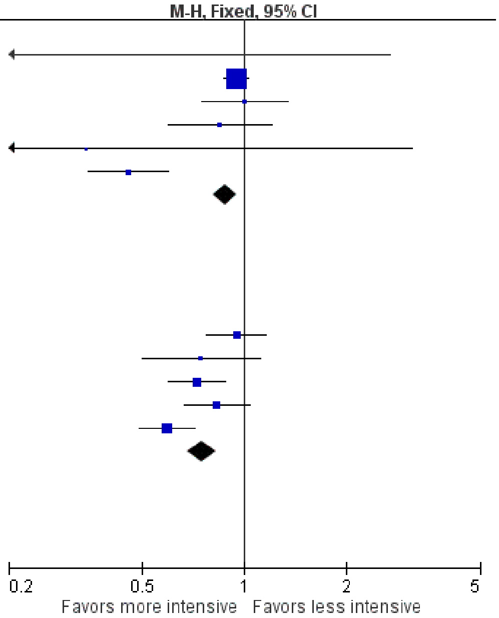

Test for subgroup differences: Chi² = 6.54, df = 1 (P = 0.01), I² = 84.7%

© 2023 Hsu CY et al. *JAMA Neurology.*

eFigure 14. Ethnicity

Legends: Relative risk with 95% confidence interval of recurrent stroke in more intensive vs less intensive blood pressure lowering in patients with stroke or transient ischemic attack in Asians vs mostly non-Asians.

	More intensive		Less intensive			Risk Ratio
Study or Subgroup	Events	Total	Events	Total	Weight	M-H, Fixed, 95% CI
**14.1.1 Asians**						
RESPECT [6]	39	633	52	630	12.4%	0.75 [0.50, 1.11]
PATS [7]	159	2840	219	2825	52.4%	0.72 [0.59, 0.88]
Liu et al [10]	67	762	147	758	35.2%	0.45 [0.35, 0.59]
**Subtotal (95% CI)**		**4235**		**4213**	**100.0%**	**0.63 [0.54, 0.73]**
Total events	265		418			
Heterogeneity: Chi² = 8.19, df = 2 (P = 0.02); I² = 76%						
Test for overall effect: Z = 6.13 (P < 0.00001)						
**14.1.2 Mostly non-Asians**						
PAST-BP [1]	0	266	3	263	0.2%	0.14 [0.01, 2.72]
PRoFESS [2]	880	10146	934	10186	56.8%	0.95 [0.87, 1.03]
TEST [3]	74	372	69	348	4.3%	1.00 [0.75, 1.35]
PROGRESS, single [4]	157	1281	165	1280	10.0%	0.95 [0.78, 1.17]
DUTCH TIA [5]	52	732	62	741	3.8%	0.85 [0.60, 1.21]
PODCAST [8]	1	41	3	42	0.2%	0.34 [0.04, 3.15]
SPS 3 [9]	125	1501	152	1519	9.2%	0.83 [0.66, 1.04]
PROGRESS, combination [4]	150	1770	255	1774	15.5%	0.59 [0.49, 0.71]
**Subtotal (95% CI)**		**16109**		**16153**	**100.0%**	**0.88 [0.82, 0.94]**
Total events	1439		1643			
Heterogeneity: Chi² = 23.32, df = 7 (P = 0.001); I² = 70%						
Test for overall effect: Z = 3.84 (P = 0.0001)						

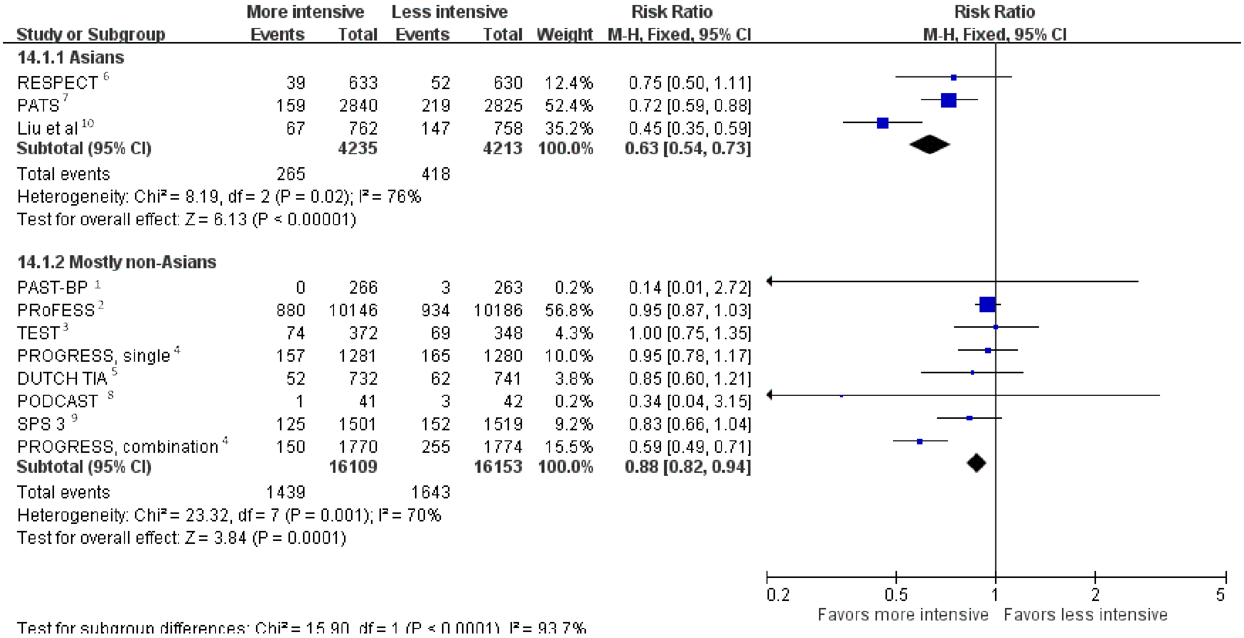

Test for subgroup differences: Chi² = 15.90, df = 1 (P < 0.0001), I² = 93.7%

© 2023 Hsu CY et al. *JAMA Neurology.*

eFigure 15. Antihypertensive drugs used in more intensive treated group

Legends: Relative risk with 95% confidence interval of recurrent stroke in more intensive vs less intensive blood pressure lowering in patients with stroke or transient ischemic attack for antihypertensive drugs used in the more intensive treated arm (angiotensin-converting enzyme [ACE] inhibitors vs angiotensin receptor blockers vs beta blockers vs diuretics vs ACI inhibitors plus diuretics).

Study or Subgroup	More intensive Events	More intensive Total	Less intensive Events	Less intensive Total	Weight	Risk Ratio M-H, Fixed, 95% CI
**15.1.1 ACE inhibitors**						
PROGRESS, single[4]	157	1281	165	1280	100.0%	0.95 [0.78, 1.17]
**Subtotal (95% CI)**		**1281**		**1280**	**100.0%**	**0.95 [0.78, 1.17]**
Total events	157		165			
Heterogeneity: Not applicable						
Test for overall effect: Z = 0.48 (P = 0.63)						
**15.1.2 Angiotensin receptor blockers**						
PRoFESS[2]	880	10146	934	10186	100.0%	0.95 [0.87, 1.03]
**Subtotal (95% CI)**		**10146**		**10186**	**100.0%**	**0.95 [0.87, 1.03]**
Total events	880		934			
Heterogeneity: Not applicable						
Test for overall effect: Z = 1.24 (P = 0.21)						
**15.1.3 Beta blockers**						
TEST[3]	74	372	69	348	53.6%	1.00 [0.75, 1.35]
DUTCH TIA[5]	52	732	62	741	46.4%	0.85 [0.60, 1.21]
**Subtotal (95% CI)**		**1104**		**1089**	**100.0%**	**0.93 [0.74, 1.17]**
Total events	126		131			
Heterogeneity: Chi² = 0.51, df = 1 (P = 0.48); I² = 0%						
Test for overall effect: Z = 0.61 (P = 0.54)						
**15.1.4 Diuretics**						
PATS[7]	159	2840	219	2825	100.0%	0.72 [0.59, 0.88]
**Subtotal (95% CI)**		**2840**		**2825**	**100.0%**	**0.72 [0.59, 0.88]**
Total events	159		219			
Heterogeneity: Not applicable						
Test for overall effect: Z = 3.23 (P = 0.001)						
**15.1.5 ACE inhibitors plus diuretics**						
PROGRESS, combination[4]	150	1770	255	1774	63.3%	0.59 [0.49, 0.71]
Liu et al[10]	67	762	147	758	36.7%	0.45 [0.35, 0.59]
**Subtotal (95% CI)**		**2532**		**2532**	**100.0%**	**0.54 [0.46, 0.63]**
Total events	217		402			
Heterogeneity: Chi² = 2.42, df = 1 (P = 0.12); I² = 59%						
Test for overall effect: Z = 7.77 (P < 0.00001)						

Test for subgroup differences: Chi² = 42.49, df = 4 (P < 0.00001), I² = 90.6%

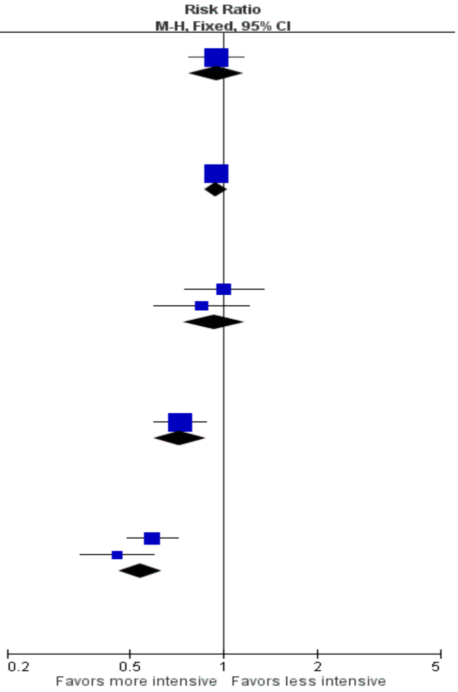

© 2023 Hsu CY et al. *JAMA Neurology.*

eFigure 16. Mean baseline SBP

Legends: Relative risk with 95% confidence interval of recurrent stroke in more intensive vs less intensive blood pressure lowering in patients with stroke or transient ischemic attack with mean baseline SBP levels ≥ 150 mmHg vs 140-149 mmHg.

SBP: systolic blood pressure

Study or Subgroup	More intensive Events	More intensive Total	Less intensive Events	Less intensive Total	Weight	Risk Ratio M-H, Fixed, 95% CI
**16.1.1 ≥ 150 mmHg**						
TEST [3]	74	372	69	348	20.2%	1.00 [0.75, 1.35]
DUTCH TIA [5]	52	732	62	741	17.5%	0.85 [0.60, 1.21]
PATS [7]	159	2840	219	2825	62.3%	0.72 [0.59, 0.88]
**Subtotal (95% CI)**		**3944**		**3914**	**100.0%**	**0.80 [0.69, 0.93]**
Total events	285		350			
Heterogeneity: Chi² = 3.42, df = 2 (P = 0.18); I² = 41%						
Test for overall effect: Z = 2.92 (P = 0.003)						
**16.1.2 140-149 mmHg**						
PAST-BP [1]	0	266	3	263	0.2%	0.14 [0.01, 2.72]
PRoFESS [2]	880	10146	934	10186	54.5%	0.95 [0.87, 1.03]
PROGRESS, single [4]	157	1281	165	1280	9.7%	0.95 [0.78, 1.17]
RESPECT [6]	39	633	52	630	3.0%	0.75 [0.50, 1.11]
PODCAST [8]	1	41	3	42	0.2%	0.34 [0.04, 3.15]
SPS 3 [9]	125	1501	152	1519	8.8%	0.83 [0.66, 1.04]
PROGRESS, combination [4]	150	1770	255	1774	14.9%	0.59 [0.49, 0.71]
Liu et al [10]	67	762	147	758	8.6%	0.45 [0.35, 0.59]
**Subtotal (95% CI)**		**16400**		**16452**	**100.0%**	**0.83 [0.78, 0.89]**
Total events	1419		1711			
Heterogeneity: Chi² = 43.95, df = 7 (P < 0.00001); I² = 84%						
Test for overall effect: Z = 5.39 (P < 0.00001)						

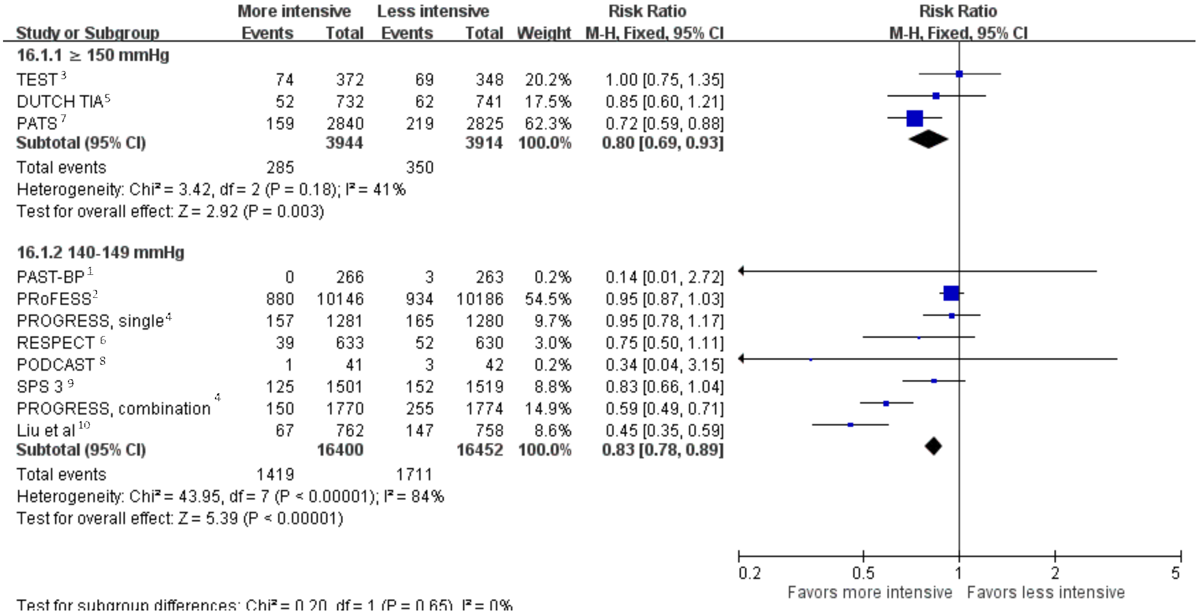

Test for subgroup differences: Chi² = 0.20, df = 1 (P = 0.65), I² = 0%

© 2023 Hsu CY et al. *JAMA Neurology.*

eFigure 17. Achieved SBP in more intensive treated group

Legends: Relative risk with 95% confidence interval of recurrent stroke in more intensive vs less intensive blood pressure lowering in patients with stroke or transient ischemic attack with achieved SBP levels in the more intensive blood pressure lowering group (≥ 140 mmHg vs 130 to < 140 mmHg vs < 130 mmHg).

SBP: systolic blood pressure

Study or Subgroup	More intensive Events	More intensive Total	Less intensive Events	Less intensive Total	Weight	Risk Ratio M-H, Fixed, 95% CI
**17.1.1 ≥ 140 mmHg**						
TEST [3]	74	372	69	348	13.8%	1.00 [0.75, 1.35]
PROGRESS, single [4]	157	1281	165	1280	31.9%	0.95 [0.78, 1.17]
DUTCH TIA [5]	52	732	62	741	11.9%	0.85 [0.60, 1.21]
PATS [7]	159	2840	219	2825	42.4%	0.72 [0.59, 0.88]
**Subtotal (95% CI)**		**5225**		**5194**	**100.0%**	**0.85 [0.75, 0.96]**
Total events	442		515			
Heterogeneity: Chi² = 5.00, df = 3 (P = 0.17); I² = 40%						
Test for overall effect: Z = 2.67 (P = 0.008)						
**17.1.2 130 to < 140 mmHg**						
PRoFESS [2]	880	10146	934	10186	78.3%	0.95 [0.87, 1.03]
PODCAST [8]	1	41	3	42	0.2%	0.34 [0.04, 3.15]
PROGRESS, combination [4]	150	1770	255	1774	21.4%	0.59 [0.49, 0.71]
**Subtotal (95% CI)**		**11957**		**12002**	**100.0%**	**0.87 [0.80, 0.94]**
Total events	1031		1192			
Heterogeneity: Chi² = 20.17, df = 2 (P < 0.0001); I² = 90%						
Test for overall effect: Z = 3.49 (P = 0.0005)						
**17.1.3 < 130 mmHg**						
PAST-BP [1]	0	266	3	263	1.7%	0.14 [0.01, 2.72]
RESPECT [6]	39	633	52	630	25.2%	0.75 [0.50, 1.11]
SPS 3 [9]	125	1501	152	1519	73.1%	0.83 [0.66, 1.04]
**Subtotal (95% CI)**		**2400**		**2412**	**100.0%**	**0.80 [0.66, 0.97]**
Total events	164		207			
Heterogeneity: Chi² = 1.55, df = 2 (P = 0.46); I² = 0%						
Test for overall effect: Z = 2.25 (P = 0.02)						

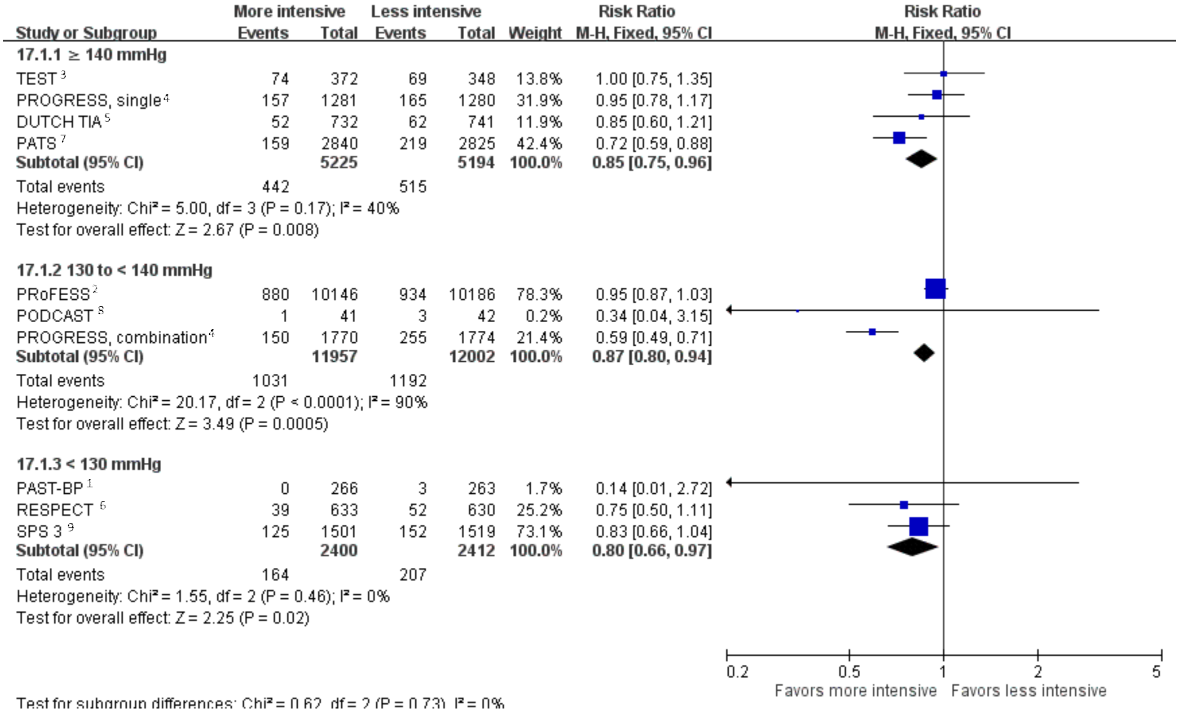

Test for subgroup differences: Chi² = 0.62, df = 2 (P = 0.73), I² = 0%

© 2023 Hsu CY et al. *JAMA Neurology.*

eFigure 18. Achieved SBP in less intensive treated group

Legends: Relative risk with 95% confidence interval of recurrent stroke in more intensive vs less intensive blood pressure lowering in patients with stroke or transient ischemic attack with achieved SBP levels in the less intensive blood pressure lowering group (≥ 140 mmHg vs 130 to < 140 mmHg vs < 130 mmHg).

SBP: systolic blood pressure

Study or Subgroup	More intensive Events	More intensive Total	Less intensive Events	Less intensive Total	Weight	Risk Ratio M-H, Fixed, 95% CI
**18.1.1 ≥ 140 mmHg**						
TEST [3]	74	372	69	348	13.8%	1.00 [0.75, 1.35]
PROGRESS, single [4]	157	1281	165	1280	31.9%	0.95 [0.78, 1.17]
DUTCH TIA [5]	52	732	62	741	11.9%	0.85 [0.60, 1.21]
PATS [7]	159	2840	219	2825	42.4%	0.72 [0.59, 0.88]
**Subtotal (95% CI)**		**5225**		**5194**	**100.0%**	**0.85 [0.75, 0.96]**
Total events	442		515			
Heterogeneity: Chi² = 5.00, df = 3 (P = 0.17); I² = 40%						
Test for overall effect: Z = 2.67 (P = 0.008)						
**18.1.2 130 to < 140 mmHg**						
PRoFESS [2]	880	10146	934	10186	66.9%	0.95 [0.87, 1.03]
RESPECT [6]	39	633	52	630	3.7%	0.75 [0.50, 1.11]
PODCAST [8]	1	41	3	42	0.2%	0.34 [0.04, 3.15]
SPS 3 [9]	125	1501	152	1519	10.8%	0.83 [0.66, 1.04]
PROGRESS, combination [4]	150	1770	255	1774	18.3%	0.59 [0.49, 0.71]
**Subtotal (95% CI)**		**14091**		**14151**	**100.0%**	**0.86 [0.80, 0.93]**
Total events	1195		1396			
Heterogeneity: Chi² = 20.80, df = 4 (P = 0.0003); I² = 81%						
Test for overall effect: Z = 4.03 (P < 0.0001)						
**18.1.3 < 130 mmHg**						
PAST-BP [1]	0	266	3	263	100.0%	0.14 [0.01, 2.72]
**Subtotal (95% CI)**		**266**		**263**	**100.0%**	**0.14 [0.01, 2.72]**
Total events	0		3			
Heterogeneity: Not applicable						
Test for overall effect: Z = 1.30 (P = 0.19)						

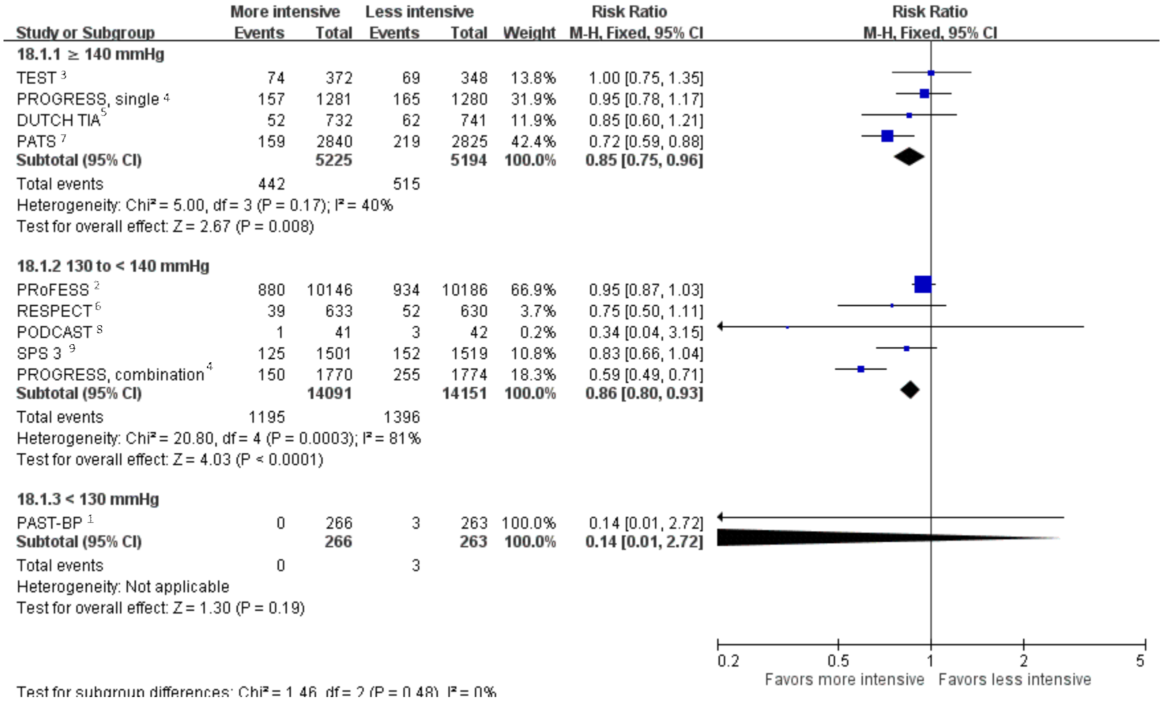

Test for subgroup differences: Chi² = 1.46, df = 2 (P = 0.48), I² = 0%

© 2023 Hsu CY et al. *JAMA Neurology.*

eFigure 19. Entry event

Legends: Relative risk with 95% confidence interval of recurrent stroke in more intensive vs less intensive blood pressure lowering in patients with stroke or transient ischemic attack with ischemic stroke vs hemorrhagic stroke as an entry event

Study or Subgroup	More intensive Events	More intensive Total	Less intensive Events	Less intensive Total	Weight	Risk Ratio M-H, Fixed, 95% CI
**19.1.1 Ischemic stroke**						
PRoFESS[2]	880	10146	934	10186	57.8%	0.95 [0.87, 1.03]
RESPECT[6]	35	532	43	542	2.6%	0.83 [0.54, 1.27]
PATS[7]	130	2127	185	2118	11.5%	0.70 [0.56, 0.87]
PROGRESS[11]	229	2135	302	2127	18.7%	0.76 [0.64, 0.89]
SPS 3[9]	125	1501	152	1519	9.4%	0.83 [0.66, 1.04]
**Subtotal (95% CI)**		**16441**		**16492**	**100.0%**	**0.87 [0.81, 0.93]**
Total events	1399		1616			
Heterogeneity: Chi² = 10.53, df = 4 (P = 0.03); I² = 62%						
Test for overall effect: Z = 4.06 (P < 0.0001)						
**19.1.2 Hemorrhagic stroke**						
RESPECT[6]	4	101	9	88	10.4%	0.39 [0.12, 1.21]
PATS[7]	28	410	29	404	31.4%	0.95 [0.58, 1.57]
PROGRESS[11]	32	306	54	305	58.2%	0.59 [0.39, 0.89]
**Subtotal (95% CI)**		**817**		**797**	**100.0%**	**0.68 [0.51, 0.92]**
Total events	64		92			
Heterogeneity: Chi² = 3.12, df = 2 (P = 0.21); I² = 36%						
Test for overall effect: Z = 2.48 (P = 0.01)						

Test for subgroup differences: Chi² = 2.31, df = 1 (P = 0.13), I² = 56.7%

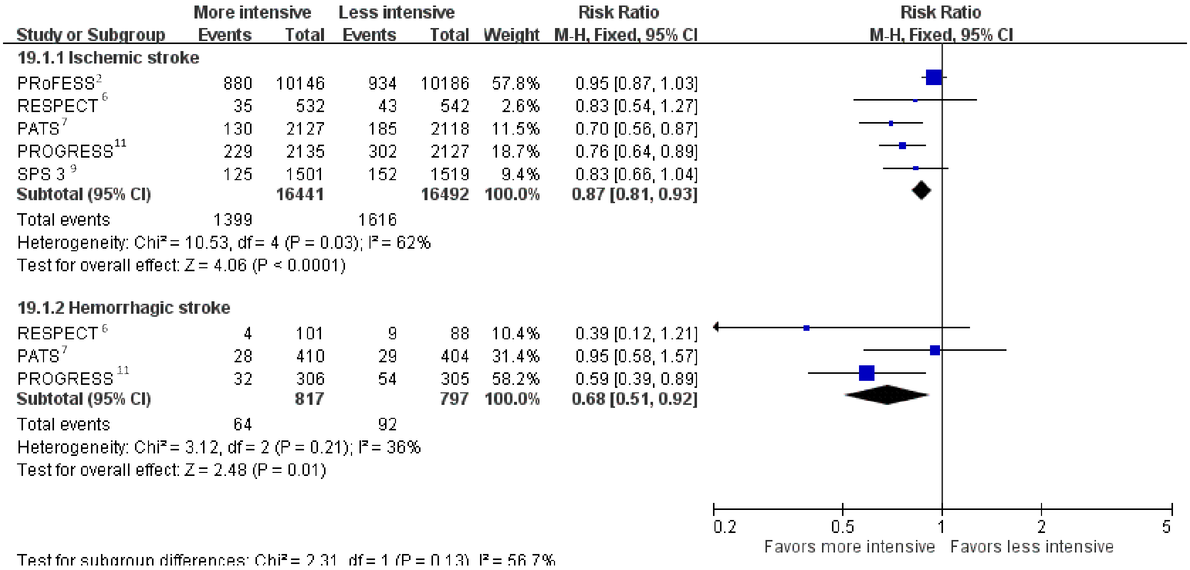

© 2023 Hsu CY et al. *JAMA Neurology.*

eFigure 20. Sample size

Legends: Relative risk with 95% confidence interval of recurrent stroke in more intensive vs less intensive blood pressure lowering in patients with stroke or transient ischemic attack with sample size < 3000 vs ≥ 3000 patients.

Study or Subgroup	More intensive Events	More intensive Total	Less intensive Events	Less intensive Total	Weight	Risk Ratio M-H, Fixed, 95% CI
**20.1.1 < 3000**						
PAST-BP [1]	0	266	3	263	0.7%	0.14 [0.01, 2.72]
TEST [3]	74	372	69	348	14.1%	1.00 [0.75, 1.35]
PROGRESS, single [4]	157	1281	165	1280	32.8%	0.95 [0.78, 1.17]
DUTCH TIA [5]	52	732	62	741	12.2%	0.85 [0.60, 1.21]
RESPECT [6]	39	633	52	630	10.3%	0.75 [0.50, 1.11]
PODCAST [8]	1	41	3	42	0.6%	0.34 [0.04, 3.15]
Liu et al [10]	67	762	147	758	29.2%	0.45 [0.35, 0.59]
**Subtotal (95% CI)**		**4087**		**4062**	**100.0%**	**0.77 [0.68, 0.87]**
Total events	390		501			
Heterogeneity: Chi² = 23.99, df = 6 (P = 0.0005); I² = 75%						
Test for overall effect: Z = 4.14 (P < 0.0001)						
**20.1.2 ≥ 3000**						
PRoFESS [2]	880	10146	934	10186	59.8%	0.95 [0.87, 1.03]
PATS [7]	159	2840	219	2825	14.1%	0.72 [0.59, 0.88]
SPS 3 [9]	125	1501	152	1519	9.7%	0.83 [0.66, 1.04]
PROGRESS, combination [4]	150	1770	255	1774	16.4%	0.59 [0.49, 0.71]
**Subtotal (95% CI)**		**16257**		**16304**	**100.0%**	**0.85 [0.79, 0.91]**
Total events	1314		1560			
Heterogeneity: Chi² = 22.47, df = 3 (P < 0.0001); I² = 87%						
Test for overall effect: Z = 4.71 (P < 0.00001)						

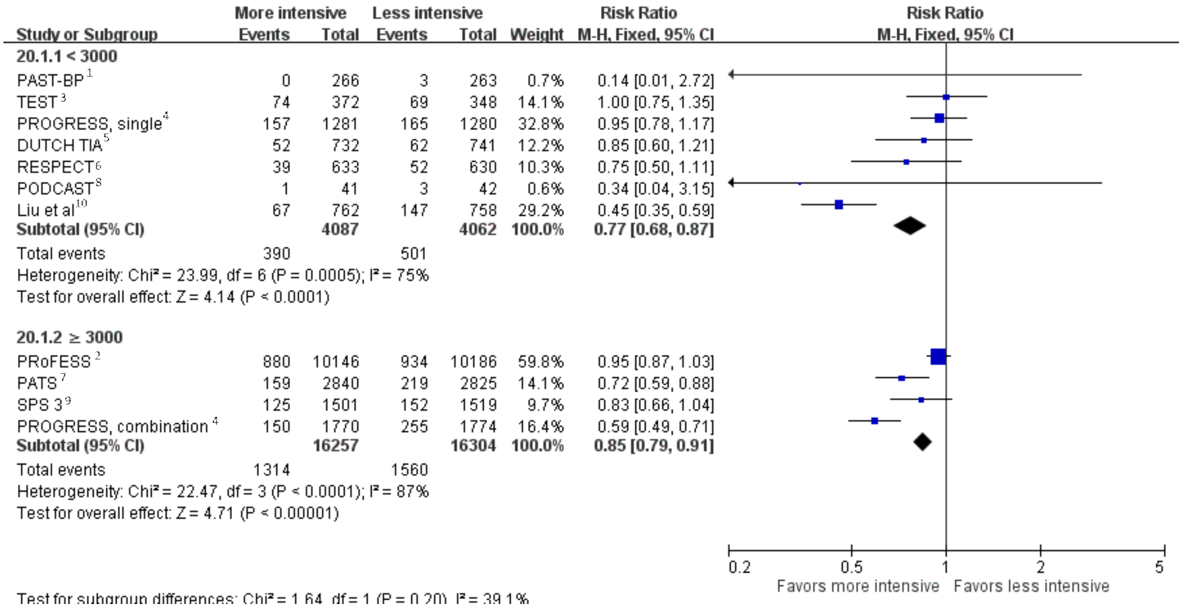

Test for subgroup differences: Chi² = 1.64, df = 1 (P = 0.20), I² = 39.1%

© 2023 Hsu CY et al. *JAMA Neurology.*

eFigure 21. Study design

Legends: Relative risk with 95% confidence interval of recurrent stroke in more intensive vs less intensive blood pressure lowering in patients with stroke or transient ischemic attack, antihypertensive drugs vs placebo and a lower blood pressure target vs a higher blood pressure target.

	More intensive		Less intensive			Risk Ratio
Study or Subgroup	Events	Total	Events	Total	Weight	M-H, Fixed, 95% CI
**21.1.1 Antihypertensive drugs vs Placebo**						
PRoFESS[2]	880	10146	934	10186	50.3%	0.95 [0.87, 1.03]
TEST[3]	74	372	69	348	3.9%	1.00 [0.75, 1.35]
PROGRESS, single[4]	157	1281	165	1280	8.9%	0.95 [0.78, 1.17]
DUTCH TIA[5]	52	732	62	741	3.3%	0.85 [0.60, 1.21]
PATS[7]	159	2840	219	2825	11.9%	0.72 [0.59, 0.88]
PROGRESS, combination	150	1770	255	1774	13.8%	0.59 [0.49, 0.71]
Liu et al[10]	67	762	147	758	8.0%	0.45 [0.35, 0.59]
**Subtotal (95% CI)**		**17903**		**17912**	**100.0%**	**0.83 [0.78, 0.89]**
Total events	1539		1851			
Heterogeneity: Chi² = 45.23, df = 6 (P < 0.00001); I² = 87%						
Test for overall effect: Z = 5.67 (P < 0.00001)						
**21.1.2 A lower BP target vs A higher BP target**						
PAST-BP[1]	0	266	3	263	1.7%	0.14 [0.01, 2.72]
RESPECT[6]	39	633	52	630	24.9%	0.75 [0.50, 1.11]
PODCAST[8]	1	41	3	42	1.4%	0.34 [0.04, 3.15]
SPS 3[9]	125	1501	152	1519	72.1%	0.83 [0.66, 1.04]
**Subtotal (95% CI)**		**2441**		**2454**	**100.0%**	**0.79 [0.65, 0.96]**
Total events	165		210			
Heterogeneity: Chi² = 2.12, df = 3 (P = 0.55); I² = 0%						
Test for overall effect: Z = 2.34 (P = 0.02)						

Test for subgroup differences: Chi² = 0.20, df = 1 (P = 0.65), I² = 0%

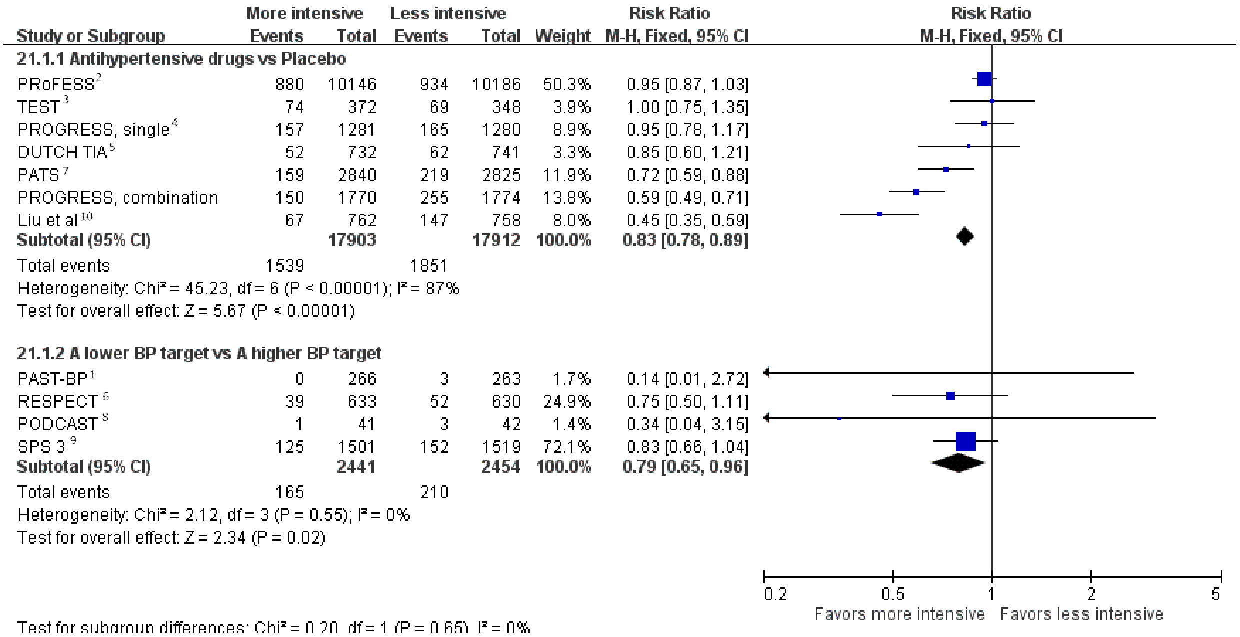

© 2023 Hsu CY et al. *JAMA Neurology.*

eFigure 22. Definition of differential blood pressure reduction

Legends: Relative risk with 95% confidence interval of recurrent stroke in more intensive vs less intensive blood pressure lowering in patients with stroke or transient ischemic attack with, average difference throughout the studies vs other definitions.

Study or Subgroup	More intensive Events	More intensive Total	Less intensive Events	Less intensive Total	Weight	Risk Ratio M-H, Fixed, 95% CI
**22.1.1 Average difference throughout the study**						
PAST-BP[1]	0	266	3	263	0.2%	0.14 [0.01, 2.72]
PRoFESS[2]	880	10146	934	10186	59.9%	0.95 [0.87, 1.03]
PROGRESS, single[4]	157	1281	165	1280	10.6%	0.95 [0.78, 1.17]
RESPECT[6]	39	633	52	630	3.4%	0.75 [0.50, 1.11]
PROGRESS, combination[4]	150	1770	255	1774	16.4%	0.59 [0.49, 0.71]
Liu et al[10]	67	762	147	758	9.5%	0.45 [0.35, 0.59]
**Subtotal (95% CI)**		**14858**		**14891**	**100.0%**	**0.83 [0.78, 0.89]**
Total events	1293		1556			
Heterogeneity: Chi² = 43.32, df = 5 (P < 0.00001); I² = 88%						
Test for overall effect: Z = 5.12 (P < 0.00001)						
**22.1.2 Other definitions**						
TEST[3]	74	372	69	348	14.1%	1.00 [0.75, 1.35]
DUTCH TIA[5]	52	732	62	741	12.2%	0.85 [0.60, 1.21]
PATS[7]	159	2840	219	2825	43.3%	0.72 [0.59, 0.88]
PODCAST[8]	1	41	3	42	0.6%	0.34 [0.04, 3.15]
SPS 3[9]	125	1501	152	1519	29.8%	0.83 [0.66, 1.04]
**Subtotal (95% CI)**		**5486**		**5475**	**100.0%**	**0.81 [0.71, 0.91]**
Total events	411		505			
Heterogeneity: Chi² = 4.05, df = 4 (P = 0.40); I² = 1%						
Test for overall effect: Z = 3.38 (P = 0.0007)						

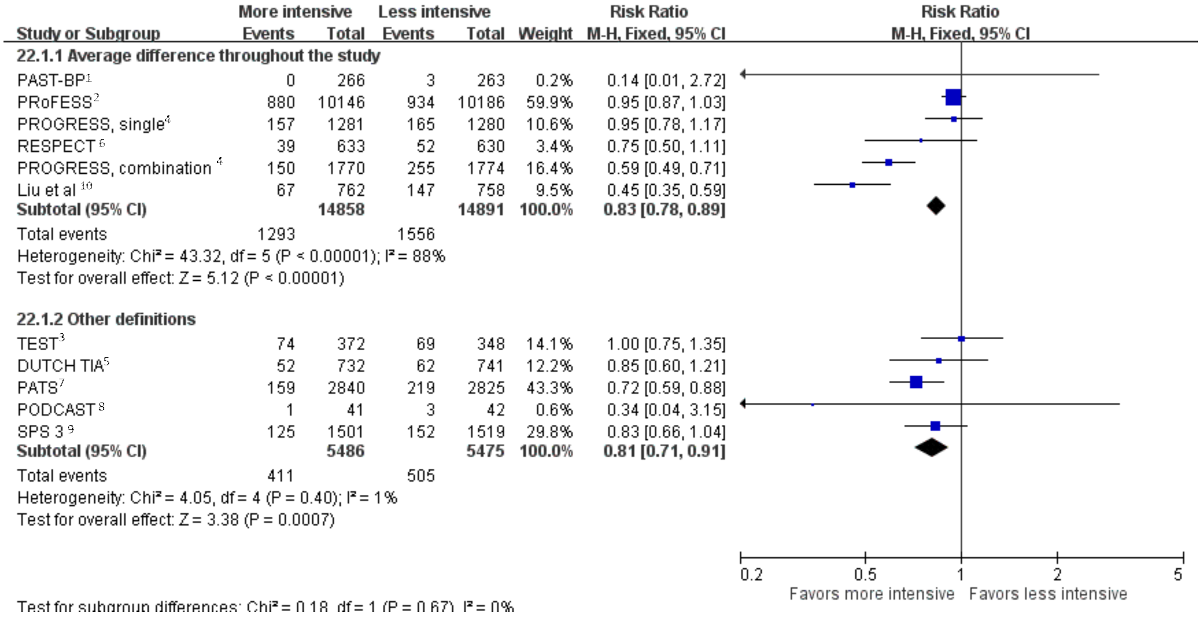

Test for subgroup differences: Chi² = 0.18, df = 1 (P = 0.67), I² = 0%

© 2023 Hsu CY et al. *JAMA Neurology.*

eFigure 23. Publication bias

Legends: Trim-and-fill analysis for included trials to explore potential publication bias

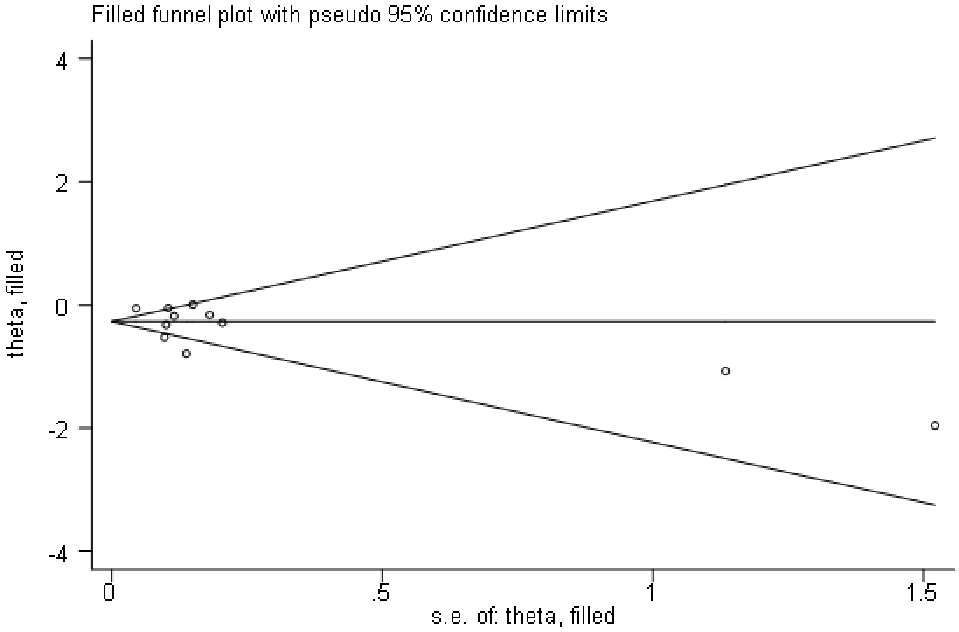

© 2023 Hsu CY et al. *JAMA Neurology.*

eReferences

1. Mant J, McManus RJ, Roalfe A, et al. Different systolic blood pressure targets for people with history of stroke or transient ischaemic attack: PAST-BP (Prevention After Stroke--Blood Pressure) randomised controlled trial. BMJ. 2016;352:i708.
2. Yusuf S, Diener HC, Sacco RL, et al. Telmisartan to prevent recurrent stroke and cardiovascular events. N Engl J Med. 2008;359(12):1225-1237.
3. Eriksson S, Olofsson B, Wesley P, the TEST study group. Atenolol in Secondary Prevention after Stroke. Cerebrovascular Diseases. 1995;5:21-25.
4. PROGRESS Collaborative Group. Randomised trial of a perindopril-based blood-pressure-lowering regimen among 6105 individuals with previous stroke or transient ischaemic attack. The Lancet. 2001;358(9287):1033-1041.
5. The Dutch TIA Trial Study Group. Trial of secondary prevention with atenolol after transient ischemic attack or nondisabling ischemic stroke. Stroke. 1993;24(4):543-548.
6. Kitagawa K, Yamamoto Y, Arima H, et al. Effect of Standard vs Intensive Blood Pressure Control on the Risk of Recurrent Stroke: A Randomized Clinical Trial and Meta-analysis. JAMA Neurol. 2019;76(11):1309-1318.
7. Liu L, Wang Z, Gong L, et al. Blood pressure reduction for the secondary prevention of stroke: a Chinese trial and a systematic review of the literature. Hypertens Res. 2009;32(11):1032-1040.
8. Bath PM, Scutt P, Blackburn DJ, et al. Intensive versus Guideline Blood Pressure and Lipid Lowering in Patients with Previous Stroke: Main Results from the Pilot "Prevention of Decline in Cognition after Stroke Trial" (PODCAST) Randomised Controlled Trial. PLoS One. 2017;12(1):e0164608.
9. The SPS3 Study Group. Blood-pressure targets in patients with recent lacunar stroke: the SPS3 randomised trial. The Lancet. 2013;382(9891):507-515.
10. Liu LS, Gong LS, Wang W, Blood Pressure Lowering to Prevent Recurrent Stroke Study G. Effects of blood pressure lowering treatment on stroke recurrence in patients with cerebrovascular diseases- a large-scale, randomized, placebo controlled trial. Zhonghua Xin Xue Guan Bing Za Zhi. 2005;33(7):613-617.
11. Chapman N, Huxley R, Anderson C, et al. Effects of a perindopril-based blood pressure-lowering regimen on the risk of recurrent stroke according

© 2023 Hsu CY et al. *JAMA Neurology.*

to stroke subtype and medical history: the PROGRESS Trial. Stroke. 2004;35(1):116-121.

© 2023 Hsu CY et al. *JAMA Neurology.*
