# Supplementary material for: Association Between Magnitude of Differential Blood Pressure Reduction and Secondary Stroke Prevention: A Meta-analysis and Meta-Regression
Source: JAMA Neurol. 2023 Mar 20;80(5):506–15. doi: 10.1001/jamaneurol.2023.0218 (PMC10028545; doi:10.1001/jamaneurol.2023.0218)
Supplement: Supplement 2. — Data sharing statement [file jamaneurol-e230218-s002.pdf]

Data Sharing Statement

Hsu. Association Between Magnitude of Differential Blood Pressure Reduction and Secondary Stroke Prevention. *JAMA Neurol*. Published March 20, 2023.
doi:10.1001/jamaneurol.2023.0218

Data
Data available: No
